# The great divide: rhamnolipids mediate separation between *P. aeruginosa* and *S. aureus*

**DOI:** 10.3389/fcimb.2023.1245874

**Published:** 2023-09-15

**Authors:** Jean-Louis Bru, Summer J. Kasallis, Rendell Chang, Quantum Zhuo, Jacqueline Nguyen, Phillip Pham, Elizabeth Warren, Katrine Whiteson, Nina Molin Høyland-Kroghsbo, Dominique H. Limoli, Albert Siryaporn

**Affiliations:** ^1^ Department of Molecular Biology & Biochemistry, University of California, Irvine, Irvine, CA, United States; ^2^ Department of Physics & Astronomy, University of California, Irvine, Irvine, CA, United States; ^3^ School of Biological Sciences, University of California, Irvine, Irvine, CA, United States; ^4^ Department of Microbiology and Immunology, University of Iowa, Iowa City, IA, United States; ^5^ Department of Plant and Environmental Sciences, University of Copenhagen, Frederiksberg, Denmark

**Keywords:** surfactant flow, swarm repulsion, tendril organization, biofilms, cystic fibrosis, rhamnolipids, structured illumination, lung surfactant

## Abstract

The interactions between bacterial species during infection can have significant impacts on pathogenesis. *Pseudomonas aeruginosa* and *Staphylococcus aureus* are opportunistic bacterial pathogens that can co-infect hosts and cause serious illness. The factors that dictate whether one species outcompetes the other or whether the two species coexist are not fully understood. We investigated the role of surfactants in the interactions between these two species on a surface that enables *P. aeruginosa* to swarm. We found that *P. aeruginosa* swarms are repelled by colonies of clinical *S. aureus* isolates, creating physical separation between the two strains. This effect was abolished in mutants of *S. aureus* that were defective in the production of phenol-soluble modulins (PSMs), which form amyloid fibrils around wild-type *S. aureus* colonies. We investigated the mechanism that establishes physical separation between the two species using Imaging of Reflected Illuminated Structures (IRIS), which is a non-invasive imaging method that tracks the flow of surfactants produced by *P. aeruginosa*. We found that PSMs produced by *S. aureus* deflected the surfactant flow, which in turn, altered the direction of *P. aeruginosa* swarms. These findings show that rhamnolipids mediate physical separation between *P. aeruginosa* and *S. aureus*, which could facilitate coexistence between these species. Additionally, we found that a number of molecules repelled *P. aeruginosa* swarms, consistent with a surfactant deflection mechanism. These include *Bacillus subtilis* surfactant, the fatty acids oleic acid and linoleic acid, and the synthetic lubricant polydimethylsiloxane. Lung surfactant repelled *P. aeruginosa* swarms and inhibited swarm expansion altogether at higher concentration. Our results suggest that surfactant interactions could have major impacts on bacteria-bacteria and bacteria-host relationships. In addition, our findings uncover a mechanism responsible for *P. aeruginosa* swarm development that does not rely solely on sensing but instead is based on the flow of surfactant.

## Introduction

*Pseudomonas aeruginosa* and *Staphylococcus aureus* are opportunistic pathogens that colonize the skin, eyes, and lungs, where they can contribute to the development of a range of illnesses (Lyczak et al., 2000; Howden et al., 2023). While a single species can dominate during infection, co-infections have been associated with worse patient outcomes (Camus et al., 2022). In particular, the two species commonly coinfect the lungs of cystic fibrosis patients (Salsgiver et al., 2016). Several factors produced by each species affect their ability to coexist. For example, 4-hydroxy-2-heptylquinoline N-oxide (HQNO), pyocyanin, and LasA protease produced by *P. aeruginosa* negatively affect *S. aureus* growth (Kessler et al., 1993; Hoffman et al., 2006; Biswas et al., 2009). Despite such factors, the species can coexist as mixed biofilms (reviewed in (Hotterbeekx et al., 2017)). In addition, recent work has revealed that *P. aeruginosa* detects and responds to the presence of *S. aureus* in several ways. For example, the presence of *S. aureus* induces exploratory motility in *P. aeruginosa* microcolonies (Limoli et al., 2019) and *P. aeruginosa* detects *S. aureus* exoproducts including intermediate metabolites and molecules that modulate the iron starvation response (Zarrella and Khare, 2022).

Additional insight into how these species interact in host environments may be gained by considering their microenvironments. *P. aeruginosa* releases several molecules into its surroundings that improve its own fitness, including rhamnolipids (RLs), siderophores, and phenazines. The production of these molecules is regulated by the cell-to-cell communication process known as quorum sensing (QS), which enables groups of bacteria to coordinate collective behaviors by emitting and detecting QS molecules (Papenfort and Bassler, 2016). RLs have a significant impact on the *P. aeruginosa* microenvironment due to their surfactant properties, ubiquity, and multifunctional roles. RLs are glycolipids that consist of rhamnose and variable-length fatty acid moieties (Jarvis and Johnson, 1949; Abdel-Mawgoud et al., 2010). They are amphipathic, containing both hydrophilic (rhamnose) and hydrophobic (fatty acid) groups, and function as surfactants, which decrease surface tension at air-liquid interfaces and interfacial tension between two liquids. Due to their surfactant properties, RLs increase the solubility of hydrophobic molecules. This property improves the ability of *P. aeruginosa* to uptake otherwise poorly soluble molecules such as hydrocarbons, which can be used as nutrient sources (Beal and Betts, 2000; Noordman and Janssen, 2002; Abdel-Mawgoud et al., 2010). RLs additionally solubilize the *P. aeruginosa* QS molecule 2-heptyl-3-hydroxy-4-quinolone (PQS) (Collier et al., 2002; Calfee et al., 2005), which could enhance PQS diffusion and sensing. RLs can directly impact *S. aureus* through the formation of micelles that transport toxins into the bacterium, altering its biofilm development and QS gene expression, and functioning as an antimicrobial (Haba et al., 2003; Benincasa et al., 2004; Gdaniec et al., 2022; Saadati et al., 2022). Serving as an important factor in pathogenesis, RLs are found in relatively high abundance in infection environments such as the lung, where they promote biofilm formation and inhibit host immunity (Kownatzki et al., 1987; McClure and Schiller, 1992; Pamp and Tolker-Nielsen, 2007; Abdel-Mawgoud et al., 2010).

RLs have a critical role in the expansion of *P. aeruginosa* swarms, which are characterized by radially expanding dendritic patterns of densely packed cells that are referred to as tendrils (Caiazza et al., 2005). Cells within swarms are highly motile via flagellar activity, grow at a high density, and have high metabolic activity (reviewed in (Kearns, 2010)). Due to the rapid expansion of swarms across large distances, swarming has been widely described as a form of motility. RLs are produced in abundance by *P. aeruginosa* in swarms, where RLs expand radially from colonies and precede tendril formation (Siegmund and Wagner, 1991; Morris et al., 2011; Xavier et al., 2011). The spatial heterogeneity and abundance of RLs present in swarms make swarming an ideal system to investigate interactions between *P. aeruginosa* and *S. aureus*.

**Figure 1:**
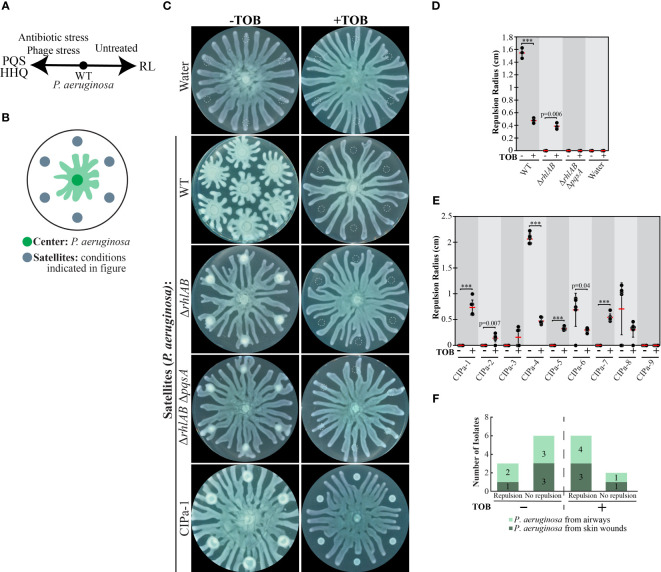
The effect of tobramycin on swarm repulsion by wild-type *P. aeruginosa*, mutants, and clinical isolates. **(A)** Schematic depicting relationship between the QS molecule 2-heptyl-3-hydroxy-4-quinolone (PQS), its precursor HHQ, and rhamnolipid (RL) production in *P. aeruginosa* on swarming plates. Untreated *P. aeruginosa* produces more RLs whereas antibiotic- or phage-stressed cells produce more PQS. **(B)** Schematic depicting swarm interaction assays in which *P. aeruginosa* is inoculated at the center and forms swarm tendrils that move outwards. **(C)** Swarm interaction assays in which wild-type (WT) *P. aeruginosa* was spotted at the center and test strains were spotted at satellite positions. Tobramycin (TOB) treatment was performed by mixing TOB with bacteria to a final concentration of 0.5 mg/mL and spotting 6 μL of the mixture onto the swarm plate. Images were acquired 18-20 hours following inoculation. Dashed lines indicate the boundaries of the initial inoculum. **(D)** Quantification of tendril repulsion by *P. aeruginosa* PA14 WT and mutant strains. Black dots represent the average radius from six satellite positions on a single plate, red bars indicate the average of three independent experiments, and error bars indicate standard deviation. **(E)** Quantification of tendril repulsion by *P. aeruginosa* clinical isolates from airways or skin wounds. Black dots represent the repulsion radius (cm) of individual satellite positions from the same plate and red bars indicate the average. Error bars indicate standard deviations. T-tests in **(D)** and **(E)** were performed as two-tailed distributions with unequal variance. *** denotes p < 0.001. T-tests in **(E)** were performed using individual satellite positions from the same plate. **(F)** Plot summarizing the number of clinical isolates that did not repel swarm tendrils (no repulsion) or exhibited repulsion at four or more out of six satellite positions (repulsion) in swarm interaction assays. Swarm assay images for the clinical isolates are shown in **Figure S2A** in the **Supplementary Materials**.

Recent work investigated the interaction between sub-populations of *P. aeruginosa* within swarms, finding that cells stressed by bacteriophage (phage) virus infection or antibiotics repel *P. aeruginosa* tendrils (Bru et al., 2019). This reorganization creates spatial separation between stressed and untreated populations, enabling “healthy” *P. aeruginosa* populations to physically avoid agents that induce stress. This collective stress response occurs through altered production of RLs, the QS-associated molecule PQS, and its precursor, 2-heptyl-4-quinolone (HHQ) (**Figure 1A**) (Bru et al., 2019). Untreated populations produce RLs that facilitate swarming. Phage or antibiotic-stressed populations increase the production of PQS, which signals the redirection of healthy tendrils away from the area of stress (Bru et al., 2019). A similar process could facilitate physical separation of *P. aeruginosa* from predators and competitors, facilitating coexistence between *P. aeruginosa* and other bacterial species. Many questions remain regarding how tendrils are steered away from PQS. For example, *P. aeruginosa* can sense PQS produced by the stressed populations through the QS receptor and transcriptional regulator PqsR (Wade et al., 2005). However, the mechanism by which the swarm alters its tendril motion in response to the detection of PQS is unknown. Additionally, it is not understood how prevalent the collective stress response is among strains of *P. aeruginosa* or other bacterial species including *S. aureus.*

*S. aureus* modifies its microenvironment in part through the production of phenol-soluble modulins (PSMs), which are amphipathic peptides that have surfactant properties, antimicrobial activity, and inhibit host innate immunity (Kaito and Sekimizu, 2007; Cogen et al., 2010; Tsompanidou et al., 2011; Peschel and Otto, 2013). Multiple PSMs have been identified, including PSMα1 to PSMα4, PSMβ1 and PSMβ2, and PSMγ, also referred to as δ-toxin (Wang et al., 2007; Peschel and Otto, 2013). δ-toxins *resemble* PSMα and are encoded by the *hld* gene. PSMs can aggregate, which results in the formation of amyloid fibrils that have multiple functions, including the fortification of the matrix of *S. aureus* biofilms and increasing the cytotoxicity of PSMs (Schwartz et al., 2012; Tayeb-Fligelman et al., 2017; Zheng et al., 2018; Zaman and Andreasen, 2020; Kreutzberger et al., 2022). PSMs facilitate the expansion of *S. aureus* on surfaces through spreading and comet formation, a mechanism similar to the active motility mechanism known as gliding (Tsompanidou et al., 2013; Pollitt et al., 2015). The functional roles of PSMs and RLs and their impact on their microenvironments suggest that they could have a significant role in *P. aeruginosa* - *S. aureus* interactions.

Here, we investigate how *P. aeruginosa* swarm tendrils interact with clinical isolates of *P. aeruginosa* and *S. aureus*. We find that clinical *P. aeruginosa* strains that are stressed by the antibiotic tobramycin repel swarm tendrils, suggesting that the collective stress response may be prevalent among *P. aeruginosa* strains. Untreated *S. aureus* repels *P. aeruginosa* swarm tendrils, an effect which is abolished in *S. aureus* strains that are defective in the production of PSMs. We use Imaging of Reflected Illuminated Structures (IRIS) (Kasallis et al., 2023) to investigate how *S. aureus* repels swarm tendrils by tracking the movement of surfactant produced by *P. aeruginosa*. We find that *S. aureus* alters the flow of surfactant from *P. aeruginosa* swarms, creating a zone of surfactant exclusion which results in tendril repulsion. This effect is significantly reduced in *S. aureus* PSM mutants. Our results show that the surfactant layer, which is composed of RLs, mediates the repulsion of *P. aeruginosa* swarms away from *S. aureus.* In support of this mechanism, multiple molecules including PQS, surfactin produced by *B. subtilis*, and lung surfactant, also repel *P. aeruginosa* swarms.

## Results

### Collective stress response in *P. aeruginosa* clinical isolates

The collective stress response was previously reported in a limited number of *P. aeruginosa* strains (Bru et al., 2019). We investigated the prevalence of the collective stress response in clinical isolates of *P. aeruginosa* using swarm interaction assays in order to determine if this response could be of clinical relevance. In these assays, *P. aeruginosa* UCBPP-PA14 was inoculated as a single spot at the center of a plate containing swarm medium and test strains were inoculated at six satellite positions surrounding the center spot (**Figure 1B**). As the center swarm expands radially, the swarm tendrils approach and interact with the strain at the satellite positions (**Figure 1B**). This swarm interaction assay enables replication of a single condition or the testing of multiple conditions on the same swarm plate. When *P. aeruginosa* strain PA14 is spotted at satellite positions, it repels the center swarm due to the production of RLs (Bru et al., 2019), an effect that is abolished in Δ*rhlAB* strains, which do not produce RLs (**Figures 1C, D**). To elicit the collective stress response, antibiotics were supplied at satellite positions at above-MIC concentrations that slow growth but do not entirely inhibit it due to the diffusion of antibiotics away from the initial spot. Previous work showed that a number of antibiotics including gentamicin, kanamycin, and fosfomycin, elicited the collective stress response, causing antibiotic-stressed strains at satellite positions to repel untreated swarms (Bru et al., 2019).

We obtained 9 clinical isolates of *P. aeruginosa* that were isolated from airway or skin infections. The isolates exhibited varying degrees of resistance to kanamycin and fosfomycin, as evidenced by variable growth among strains that were treated with these antibiotics (**Figure S1** in **Supplementary Materials** and (Bru et al., 2019)). In contrast, the strains were largely sensitive to tobramycin (TOB), as evidenced by the inhibition of growth or swarming in all but one TOB-treated satellite colonies (**Figures 1C, E, F** and **Figure S2A** in **Supplementary Materials**). TOB targets the bacterial 30S ribosomal subunit, blocks tRNA translocation (Ying et al., 2019), and induces the production of PQS in *P. aeruginosa* (Morales-Soto et al., 2018; Rieger et al., 2020). TOB-treated WT and Δ*rhlAB* strains at satellite positions were significantly inhibited for growth but repelled the untreated center swarm (**Figures 1C, D**). This result is consistent with TOB triggering the PQS-mediated collective stress response. We note that PQS alone is sufficient to repel the center swarm ((Bru et al., 2019) and **Figure S2B** in **Supplementary Materials**). TOB treatment of *P. aeruginosa* Δ*rhlAB* Δ*pqsA*, which lacks the ability to produce both RLs and PQS, failed to elicit tendril repulsion (**Figures 1C, D**), further supporting the dependence of the TOB-induced repulsive response on PQS. Center swarm tendrils collided with satellite positions containing only TOB, indicating that the repulsion is not due to the presence of TOB alone (**Figures 1C, D**). Together, the results suggest that TOB treatment of *P. aeruginosa* stimulates the collective stress response and repels swarms through the production of PQS. These results are consistent with the model that *P. aeruginosa* swarms produce RLs in non-stressed conditions but increase PQS production under stress from antibiotics (**Figure 1A**).

We assessed the TOB-induced stress response in the clinical isolates of *P. aeruginosa* (CIPa). Colonies were considered to possess at least partial ability to repel the center swarm if the tendrils did not come into contact with four or more of the satellite inoculum positions. In the absence of TOB, three of the clinical isolates (CIPa-4, CIPa-6, CIPa-8) repelled the center swarm (**Figures 1E, F** and **Figure S2A** in **Supplementary Materials**). We attribute the variability in repulsion among the clinical isolates to variations in swarm phenotypes, which are affected by the production of RLs and PQS in these strains. Treatment with TOB significantly reduced *P. aeruginosa* growth and caused at least partial repulsion of the swarm tendrils in 7 out of the 9 clinical isolates (**Figures 1E, F** and **Figure S2A** in **Supplementary Materials**). The only strains that repelled at less than four out of six satellite positions when treated with TOB were CIPa-3 and CIPa-9, the latter of which appeared to be TOB-resistant. The significant increase in the number of strains that repelled swarms due to TOB treatment (**Figure 1F**) is consistent with activation of a collective stress response and is consistent with a model in which PQS is produced in response to antibiotic stress in the TOB-sensitive clinical isolates tested. Together, these results suggest that the activity of the collective stress response is present in *P. aeruginosa* clinical isolates that cause disease in human airways and skin wounds.

### *S. aureus* clinical isolates repel *P. aeruginosa* swarms

**Figure 2:**
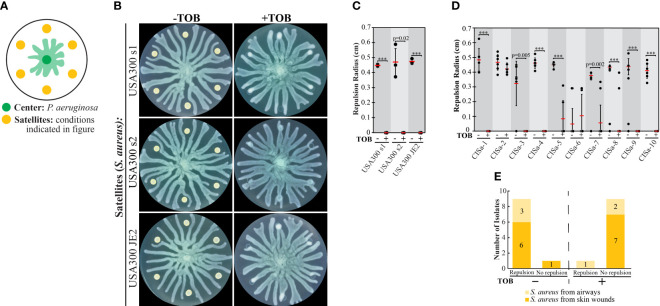
The effect of tobramycin on swarm repulsion by wild-type *S. aureus* and clinical isolates. **(A)** Schematic indicating swarm interaction assay in which *P. aeruginosa* and *S. aureus* are inoculated at the center and satellite positions respectively, and *P. aeruginosa* swarm tendrils move outward towards the satellite positions. **(B)** Swarm interaction assays in which WT *P. aeruginosa* and WT *S. aureus* strains were spotted at satellite positions. TOB treatment was performed by mixing TOB with bacteria to a final concentration of 0.5 mg/mL and spotting 6 μL of the mixture onto the swarm plate. Plates were imaged 18-20 hours following inoculation. **(C)** Quantification of repulsion by *S. aureus* USA300 strains. Black dots represent the average repulsion radius from six satellite positions, red bars indicate the average of three independent experiments, and error bars indicate standard deviation. **(D)** Quantification of repulsion by *S. aureus* clinical isolates from airways or skin wounds. Black dots represent the repulsion radius of individual satellites from the same plate and red bars indicate the average. Error bars indicate standard deviation. T-tests in **(C, D)** were performed as two-tailed distributions with unequal variance. *** denotes p < 0.001. **(E)** Plot summarizing the number of clinical isolates that did not repel swarm tendrils (no repulsion) or exhibited repulsion at four or more out of six satellite positions (repulsion) in swarm interaction assays. Swarm assay images for the clinical isolates are shown in **Figure S3A** in the **Supplementary Materials**.

The collective stress response in *P. aeruginosa* could promote its survival in natural environments by separating the swarm population from areas that contain phage or antibiotics. We reasoned that *P. aeruginosa* could use a similar strategy to isolate itself from other bacterial species. We assessed the interaction between *P. aeruginosa* and *S. aureus* by inoculating *P. aeruginosa* at the swarm plate center and *S. aureus* at satellite positions (**Figure 2A**). Under these conditions, *P. aeruginosa* developed tendrils that moved outwards whereas *S. aureus* grew as colonies that did not expand spatially. We tested the interaction of *P. aeruginosa* with multiple methicillin-resistant *S. aureus* USA300 (LAC) clinical isolates, which we refer to as USA300 s1, USA300 s2, and USA300 JE2. These strains strongly repelled *P. aeruginosa* tendrils (**Figures 2B, C**), similar to antibiotic-stressed *P. aeruginosa*. The treatment of these *S. aureus* strains with TOB reduced colony growth but did not completely abolish it, similar to the effect on *P. aeruginosa* strains (**Figures 1C**, **2B**). However, TOB treatment of *S. aureus* suppressed the repulsion of *P. aeruginosa* tendrils and enabled the tendrils to infiltrate the *S. aureus* colonies (**Figures 2B, C**). TOB treatment of the clinical strains of *S. aureus* (CISa), which were isolated from airways and skin wounds, largely reduced colony growth (**Figure S3A** and **Movie S1** in **Supplementary Materials**). Growth was quantified for CISa strains for which the growth was difficult to discern visually and confirmed that TOB did not abolish growth (**Figure S3B** in **Supplementary Materials**). Similar to the USA300 strains, untreated CISa strains largely repelled *P. aeruginosa* tendrils (9 out of the 10 clinical isolates) and TOB treatment largely suppressed tendril repulsion (**Figures 2D, E** and **Figure S3A** in **Supplementary Materials**).

Whereas TOB treatment of *P. aeruginosa* caused tendril repulsion, this treatment of *S. aureus* suppressed tendril repulsion. Thus, *P. aeruginosa* is repelled by antibiotic stress of its own species, but not by antibiotic stress of its competitor *S. aureus*. This result suggests that the collective stress response is beneficial for only its own species. These findings raise the question of how *S. aureus* repels *P. aeruginosa* tendrils. While tendrils are repelled by antibiotic-stressed *P. aeruginosa* via the production of the QS molecule PQS, *S. aureus* does not contain the genes necessary for PQS synthesis. We hypothesized that *S. aureus* produces another QS-associated molecule that alters tendril direction.

### PSM fibrils facilitate repulsion of *P. aeruginosa* swarms

**Figure 3:**
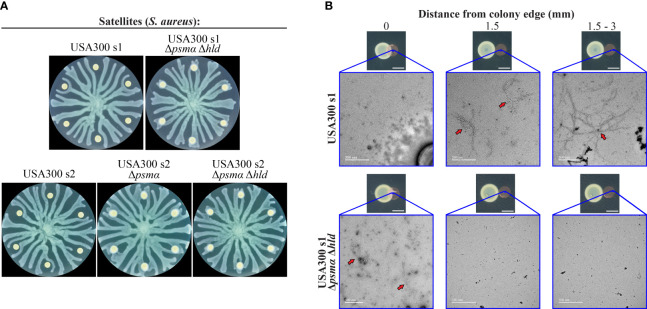
Dependence of *P. aeruginosa* tendril repulsion on phenol-soluble modulins (PSMs). **(A)** Swarm interaction assays in which WT *P. aeruginosa* was spotted at the center and WT *S. aureus* strains (USA300 s1 and s2) or their respective Δ*psmα* or Δ*psmα* Δ*hld* mutants were spotted at the satellite positions. The images for WT *S. aureus* are the same as those in **Figure 2B** and are shown here for reference. **(B)** Copper grids were placed at the same location as or adjacent to inoculums of WT *S. aureus* or the Δ*psmα* Δ*hld* mutant on swarm plates and incubated 18 to 20 hours (small images). Grids were imaged using transmission electron microscopy (TEM) (large images). Fibrils (red arrows) were observed at all positions on the grids in the vicinity of the WT *S. aureus* colony. No fibrils were observed at positions that were 1.5 mm or greater from the edge of the *S. aureus* Δ*psmα* Δ*hld* colony. Scale bars indicate 3 mm and 500 nm in the small and large images, respectively.

We investigated the potential role of the QS-regulated PSMs produced by *S. aureus* in the repulsion of *P. aeruginosa* tendrils. We assessed the extent of repulsion by the *S. aureus* Δ*psmα* mutant. Whereas WT *S. aureus* strongly repelled *P. aeruginosa* swarms (**Figures 2B, C**), the Δ*psmα* mutant was deficient in repulsion, with *P. aeruginosa* tendrils infiltrating these colonies (**Figure 3A**). The USA300 Δ*psmα* Δ*hld* mutant, which does not produce PSMα or the δ-toxin, were also deficient in repelling *P. aeruginosa* swarms (**Figure 3A**). These findings suggest that PSMα peptides significantly contribute to the repulsion of *P. aeruginosa* swarms.

*S. aureus* PSMs can form amyloid fibrils that support biofilm structures (Tayeb-Fligelman et al., 2017; Zheng et al., 2018; Zaman and Andreasen, 2020; Kreutzberger et al., 2022). We considered the possibility that PSM fibrils could mediate *P. aeruginosa* repulsion. First, we investigated the formation of fibrils by PSMs on the surface of the swarm plate surface using transmission electron microscopy (TEM). Carbon-coated copper TEM grids were placed at or adjacent to *S. aureus* inoculation sites at satellite positions and were incubated for the same period of time as the swarming assays to allow for PSM production and spreading. In WT *S. aureus*, we observed fibrous structures that were consistent with amyloid fibrils. The fibrils were present at distances up to 2.5 mm from the colony edge (**Figure 3B** and **Figure S4A** in **Supplementary Materials**). In contrast, no fibrils were observed in the Δ*psmα* Δ*hld* mutant beyond the colony edge (**Figure 3B** and **Figure S4B** in **Supplementary Materials**). These observations suggest that PSMs form a layer of amyloid fibrils that surround WT *S. aureus* colonies.

### Deflection of the surfactant layer causes tendril repulsion

To uncover the mechanism underlying the repulsion of tendrils by PSM amyloid fibrils, we considered two models that could explain tendril repulsion by PSMs: (1) cellular sensing of the PSMs and (2) a physicochemical mechanism that alters the development of tendrils. In the first model, *P. aeruginosa* could sense PSM fibrils and alter its motility away from them. For example, the *P. aeruginosa* pilus chemoreceptor PilJ is involved in sensing *S. aureus* and it is possible that it could detect PSMs (Yarrington et al., 2022). In the second model the repulsion of tendrils does not require detection by *P. aeruginosa*, but is due to physicochemical interactions between the amphipathic PSMs and the swarm. In this model, PSMs form a fibril boundary around *S. aureus* that repels approaching tendrils by altering the spatial distribution of the RLs near the *P. aeruginosa* tendrils.

**Figure 4:**
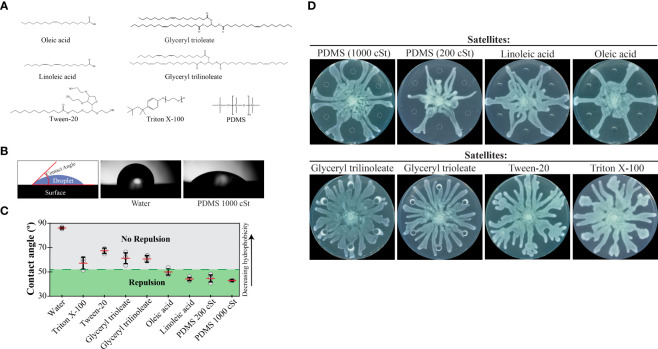
Repulsion of *P. aeruginosa* swarms by hydrophobic molecules. **(A)** Chemical structures of hydrophobic molecules that were tested for repulsion in swarm interaction assays. **(B)** Schematic indicating how contact angles were measured using images from a contact angle goniometer. Images of droplets on an oleophobic surface are shown for water and 1000 cSt PDMS, which gave the highest and lowest contact angles, respectively. **(C)** Contact angle measurements for each of the molecules. Repulsion or lack of repulsion was assessed using the data in **Figures 4D** and **1C**. Points indicate the average measurement of the left and right sides of each droplet. Red lines indicate the average (n=6) and error bars indicate standard deviation. Representative droplet images can be found in **Figure S5A** in the **Supplementary Materials**. **(D)** Swarm interaction assays in which WT *P. aeruginosa* and test molecules were spotted at the center or satellite positions, respectively. Images were acquired 15 hours following inoculation. Triton X-100 and Tween-20 were used at concentrations of 0.2% and 2%, respectively, due to their inhibitory effect on *P. aeruginosa* growth at higher concentrations.

To differentiate between these two models and better understand tendril repulsion, we performed a series of experiments using molecules with a range of hydrophobicities. We hypothesized that hydrophobicity would be an important determinant in tendril repulsion. We selected non-polar molecules with low volatilities to ensure that they remained on the swarm plate throughout the duration of the assays. Long-chain carbon molecules fit these criteria, including: oleic acid and linoleic acid (fatty acids), glyceryl trioleate and glyceryl trilinoleate (triglyceride forms of the fatty acids), Triton X-100 and Tween-20 (surfactants), and the synthetic liquid lubricant polydimethylsiloxane (PDMS) (**Figure 4A**).

The relative hydrophobicity of each molecule was determined by measuring its contact angle using a contact angle goniometer. This device measures the angle that a droplet makes with the surface and gives a quantitative measure of hydrophobicity (**Figure 4B**). We formed droplets on an oleophobic surface to maintain droplet forms for a wide range of hydrophobicities using a single surface. PDMS had the smallest contact angle, indicating the greatest hydrophobicity of the molecules tested (**Figure 4C**). In order of decreasing hydrophobicity (increasing contact angle) were PDMS, the fatty acids, triglycerides, and the surfactant Tween-20 (**Figure 4C** and **Figure S5** in **Supplementary Materials**). Water was included as a reference and had the greatest contact angle. We spotted 6 µL of each molecule at satellite positions in swarm interaction assays. In support of our hypothesis, PDMS and the fatty acids repelled tendrils (**Figure 4D**). Triglycerides and surfactants, which are less hydrophobic, did not repel tendrils (**Figure 4D**). In addition, we tested the effect of viscosity on tendril repulsion by using PDMS at two different viscosities, 200 cSt and 1000 cSt. For reference, water has a viscosity of 1 cSt at 20°C. The lower viscosity (200 cSt) PDMS produced greater repulsion than the higher viscosity PDMS (1000 cSt) (**Figure 4D** and **S5B** in **Supplementary Materials**), suggesting that a lower viscosity molecule of the same hydrophobicity produces greater repulsion. Together, these results show that hydrophobic molecules repel swarm tendrils.

**Figure 5:**
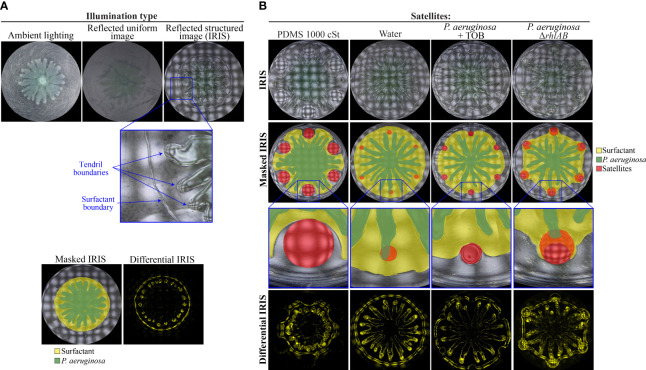
Role of the *P. aeruginosa* surfactant layer in swarm repulsion. **(A)** Swarm assay in which WT *P. aeruginosa* was spotted at the center and imaged using ambient lighting, a reflected uniform image, or a reflected structured image at 11 hours following inoculation. The enlarged portion of the IRIS Image shows tendril boundaries and surfactant boundary. The masked IRIS image indicates the surfactant layer (yellow) and *P. aeruginosa* (green) boundaries. The differential IRIS image indicates components of the surfactant layer and *P. aeruginosa* that are dynamic. **(B)** IRIS, masked IRIS, and differential IRIS images of swarm interaction assays in which WT *P. aeruginosa* was spotted at the center and either test molecules (1000 cSt PDMS or water) or strains (TOB-treated *P. aeruginosa* or *P. aeruginosa* Δ*rhlAB*) were spotted at satellite positions. Images were acquired 12-14 hours following inoculation. Masked IRIS images indicate the surfactant layer (yellow), *P. aeruginosa* (green), and test molecules or strains at the satellite positions (red). In Δ*rhlAB*, the surfactant layer overlaps the Δ*rhlAB* satellite colonies. Dashed lines at satellite positions show the boundary of the initial spot. Magnified raw IRIS images that are not masked can be found in **Figure S6C** in the **Supplementary Materials**.

We next hypothesized that hydrophobic molecules repel tendrils by altering the spatial organization of surfactant that is produced by the swarm. The surfactant contains RLs and precursor 3-(3-hydroxyalkanoyloxy) alkanoic acids (HAAs), which facilitate tendril development (Caiazza et al., 2005; Soberón-Chávez et al., 2005; Abdel-Mawgoud et al., 2010). Tracking the spatial distribution of surfactants has been a significant challenge because they are optically transparent. Surfactants from swarms have been visualized using methylene blue and Nile red dyes (Siegmund and Wagner, 1991; Morris et al., 2011) but these dyes can alter surfactant and swarm dynamics. To better measure the dynamics of surfactants and swarm tendrils without perturbing them, we used the recently-developed imaging method IRIS, which requires no modification of growth conditions or staining (Kasallis et al., 2023). IRIS applies the principle of deflectometry to illuminate swarm surfaces by projecting a structured image onto the surface and capturing the reflection (**Figure S6A**). The structured image increases the contrast of surface features, most notably the liquid-air interface at the edge of the swarm. Conventional ambient lighting and reflection of a non-structured image capture the boundaries of tendrils but do not differentiate the surfactant from the surrounding surface (**Figure 5A**). In contrast, IRIS imaging reveals high resolution details about the swarm. Notably, the tendrils grow on the surface of an optically transparent liquid-like zone (**Figure 5A**). We used edge detection to further demarcate the tendrils and the boundary of the liquid-like zone, resulting in masked IRIS images (**Figure 5A**). The liquid-like zone is not present in the Δ*rhlAB* mutant, which does not produce the HAAs or RLs (**Figure S6B** in **Supplementary Materials**). This observation suggests that the liquid-like zone is composed of HAAs and RLs.

Recent work using shadowgraphy and optical profilometry revealed that RLs penetrate the agar layer, causing the agar to swell (Deforet, 2023). This effect alters the surface of the agar such that the RL-swelled agar is raised relative to the surrounding agar surface and *P. aeruginosa* is present above the RL-swelled agar ((Deforet, 2023) and **Figure 5A**). The liquid-like zone observed using IRIS is consistent with the penetration of RLs throughout the agar and the presence of RLs at the surface of the agar. It is unclear whether RLs at the surface are suspended in water, are present as a slurry of RL with agar, or are contained within the swelled agar. We refer to the liquid-like zone observed here as the surfactant layer because it requires HAAs and RLs and appears below the tendrils. We performed differential analysis of timelapse IRIS images to further demarcate tendril and surfactant layer boundaries and identify the components of the swarm that were dynamic (**Figure 5A**). A previous analysis showed that the surfactant layer expands in lockstep with the tendril edges, which suggests that movement of the surfactant layer and tendrils are coupled (Kasallis et al., 2023). Building upon this finding, we considered the possibility that hydrophobic molecules could alter the flow of the surfactant layer, which in turn could alter the direction of tendrils.

We imaged the dynamics of the *P. aeruginosa* surfactant layer and its interaction with the hydrophobic molecules using IRIS timelapse imaging. PDMS, which was the most hydrophobic molecule that we tested (**Figure 4C**), strongly deflected the surfactant layer, causing the surfactant to flow clearly around the PDMS (**Figure 5B**, and **Figure S6C** and **Movie S2** in **Supplementary Materials**). Concomitant with the deflection of the surfactant, the tendrils moved around the PDMS. A notable feature of the dynamics is that the tendrils were constrained to move within the boundary of the surfactant layer. These observations raise the possibility that the surfactant layer sets the boundaries on which the tendrils can move, which constrains and alters the tendril movements. It is unlikely that *P. aeruginosa* could alter the direction of the surfactant flow to cause the surfactant to move around the PDMS. A more plausible explanation is that the hydrophobicity of the PDMS deflects the surfactant layer, causing the surfactant to move around the PDMS. Consistent with this, neither the surfactant layer nor tendrils were deflected by water (**Figure 5B** and **Figure S6C** in **Supplementary Materials**). These results suggest that the physicochemical interaction between the surfactant layer and PDMS is responsible for tendril repulsion by PDMS, though the interpretation does not entirely rule out a role for sensing of PDMS by *P. aeruginosa*. In support of the physicochemical interpretation, oleic acid and linoleic acid formed large boundaries that could contribute to the deflection of the surfactant layer (**Figure S6D**).

We hypothesized that stressed *P. aeruginosa* populations (**Figures 1C, D**) repel tendrils through surfactant deflection. In support of this hypothesis, surfactant from the *P. aeruginosa* swarm was deflected by TOB-treated *P. aeruginosa* satellite populations (**Figure 5B**). The movement of swarm tendrils away from TOB-treated populations was concomitant with the deflection of the surfactant (**Movie S3** in **Supplementary Materials**). These data suggest that tendril repulsion by antibiotic-stressed *P. aeruginosa* is caused by deflection of the surfactant layer, which in turn, alters the movement of swarm tendrils around the stressed *P. aeruginosa* population. We verified that *P. aeruginosa* alone does not repel tendrils or deflect the surfactant layer by using the Δ*rhlAB* strain, which is deficient in RL production and in these growth conditions produces insufficient PQS to repel tendrils without antibiotic stress (Bru et al., 2019). Surfactant produced by the WT swarm first merged with Δ*rhlAB* colonies at the satellite positions, followed by the advancement of swarm tendrils into the satellite boundaries (**Figure 5B** and **Figure S6C** in the **Supplementary Materials**).

The effects on the surfactant layer were prominent in differential IRIS images, which show that the surfactant boundary formed a concave feature as it approached PDMS or TOB-treated *P. aeruginosa* (**Figure 5B**). In contrast, no concave features were observed in the surfactant layer as it approached the Δ*rhlAB* mutant colony. In addition, the surfactant was attracted to the Δ*rhlAB* colony, causing the surfactant layer to develop into a hexagonal pattern (**Figure 5B** and **Movie S4** in **Supplementary Materials**). We note that surfactant flow from the WT swarm is observed within the boundary of the Δ*rhlAB* satellite colony, as detected by the differential IRIS images. This flow is not observed in the TOB-treated WT colonies (**Movie S3** in **Supplementary Materials**) and is consistent with merging of the surfactant layer with the Δ*rhlAB* colony.

**Figure 6:**
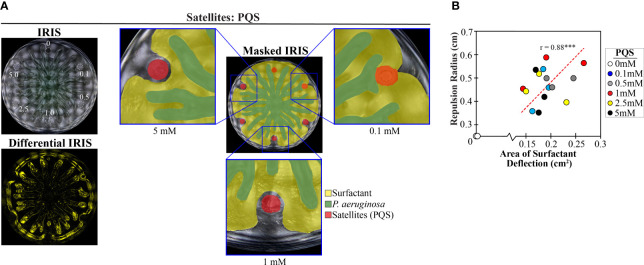
PQS increases both surfactant deflection and tendril repulsion. **(A)** IRIS, masked IRIS, and differential IRIS images of a swarm interaction assay in which WT *P. aeruginosa* was spotted at the center and a range of PQS concentrations (indicated in mM) was spotted at satellite positions. Dashed lines in the IRIS image indicate the boundaries of the initial spots. Masked IRIS images indicate surfactant layer (yellow), *P. aeruginosa* (green), and initial PQS spots (red). The differential IRIS image indicates components of the surfactant layer and *P. aeruginosa* that are dynamic. Magnified raw IRIS images that are not masked are shown in **Figure S6C** in the **Supplementary Materials**. **(B)** Tendril repulsion radii and surfactant deflection areas for the range of PQS concentrations from three independent experiments. The correlation coefficient (r value) and a least squares fit (dashed line) are displayed on the plot. *** denotes p < 0.001 computed using a two-tailed t-test. Repulsion radius was measured as the distance from the nearest tendril to the center of the PQS spot, regardless of whether the tendril contacted the boundary of the initial spot. Images were acquired 14.5-17 hours following inoculation.

We reasoned that PQS could deflect the surfactant layer from approaching tendrils, based on the observation that PQS repels swarm tendrils ((Bru et al., 2019) and **Figure S2B**). Indeed, IRIS imaging showed that PQS deflects the surfactant layer, causing a simultaneous change in tendril direction (**Figure 6A** and **Movie S5** in **Supplementary Materials**). Dimethyl sulfoxide (DMSO), the solvent in which PQS was dissolved, did not deflect the surfactant layer (**Figure S7A** in **Supplementary Materials**). Concave features were observed in the surfactant layer in the differential IRIS image, consistent with the surfactant deflection that was observed with PDMS and TOB-treated *P. aeruginosa* (**Figure 5B**). We measured the magnitude of surfactant deflection by PQS through image analysis (see Methods section and **Figure S7B** in **Supplementary Materials**) and found that the surfactant deflection area and tendril repulsion radius both increased with increasing concentrations of PQS (r = 0.88) (**Figure 6B**). This finding supports the hypothesis that PQS repels tendrils physically by disrupting the surfactant layer flow. Together, these data suggest that tendril repulsion by stressed *P. aeruginosa* populations is caused by physicochemical interactions between PQS and the surfactant layer. These interactions in turn alter the surfactant flow and tendril direction around stressed *P. aeruginosa* populations. We note that while surfactant layer deflection has a significant role in tendril repulsion, the data do not rule out a role for sensing of PQS by the *P. aeruginosa* cells within the tendrils. It is possible that PQS sensing could affect the surfactant production or composition that could indirectly affect tendril direction.

### *S. aureus* repels *P. aeruginosa* tendrils by deflecting the surfactant layer

**Figure 7:**
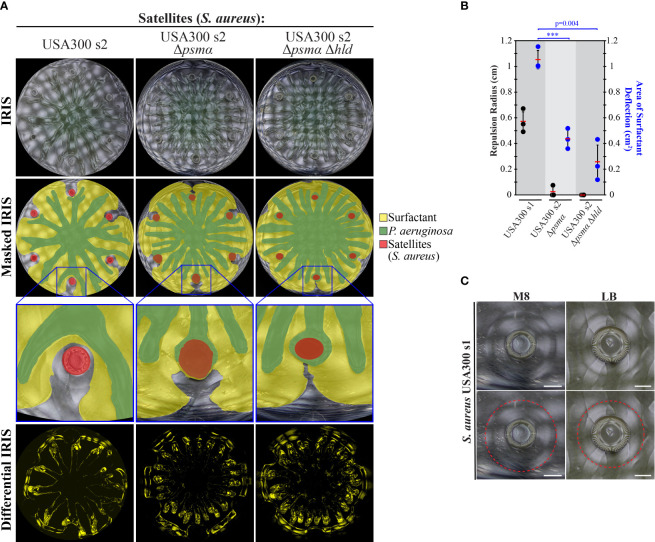
Surfactant layer deflection is diminished in PSM mutants. **(A)** IRIS, masked IRIS, and differential IRIS images of swarm interaction assays in which WT *P. aeruginosa* was spotted at the center and WT *S. aureus* or PSM mutants were spotted at satellite positions. The masked IRIS images indicate the surfactant layer (yellow), *P. aeruginosa* (green), and initial spots (red). Differential IRIS images indicate components of the surfactant layer and *P. aeruginosa* that are dynamic. Magnified raw IRIS images that are not masked are shown in **Figure S6C** in the **Supplementary Materials**. **(B)** Tendril repulsion radii (black dots) and surfactant deflection area (blue dots) of WT *S. aureus* and PSM mutants. The dots represent the average radius or area from six satellite positions on a single plate, red bars indicate the average of three independent experiments, and error bars indicate standard deviation. T-tests were performed as two-tailed distributions with unequal variance. *** denotes p < 0.001. **(C)** IRIS images of the fluidic boundary produced by *S. aureus* USA300 s1 cultured on M8 or LB medium containing 0.5% agar. The fluidic boundary is outlined in the lower images (red dashed line). Scale bar represents 3 mm. Images were acquired 14 hours following inoculation.

We rationalized that the repulsion of tendrils by *S. aureus* could be caused by a similar repulsion mechanism observed by PDMS, TOB-treated *P. aeruginosa*, and PQS. In particular, the amphipathic property of the PSM fibrils produced by *S. aureus* could deflect the *P. aeruginosa* surfactant layer, thereby altering tendril direction. IRIS imaging revealed that the *P. aeruginosa* surfactant layer was indeed deflected and moved around WT *S. aureus* colonies (**Figures 7A, B**, **Figure S6C** and **Movie S6** in **Supplementary Materials**). Surfactant deflection was significantly reduced in Δ*psmα* and Δ*psmα* Δ*hld* mutants (**Figures 7A, B**), which are defective in the production of PSMs and do not repel tendrils. These data suggest that PSMs produced by *S. aureus* deflect the surfactant layer, which results in tendril repulsion.

Additional support for surfactant deflection by *S. aureus* is the observation of a fluidic boundary that moves outwards from WT *S. aureus* colonies during growth. The fluidic boundary surrounds the colony and extends 3 mm beyond the colony edge (**Figure 7C** and **Movie S6** in **Supplementary materials**). While the composition of the fluidic boundary is unclear, we observed amyloid fibrils at a comparable distance (1.5 to 3 mm) from the *S. aureus* colony using TEM (**Figure 3B**). The observation of the fluidic boundary at a comparable distance as the fibrils offers a potential explanation for how the fibrils could be transported away from the colony edge. The fibrils are absent at this distance in the Δ*psmα* Δ*hld* mutant (**Figure 3B**), which is consistent with the model that PSM fibrils mediate the deflection of the *P. aeruginosa* surfactant layer. We note that concave features in the surfactant layer were observed in the differential IRIS images near WT *S. aureus* colonies (**Figure 7A**). The features are similar to the concave features produced by other tendril-repelling molecules (**Figures 5B**, **6A**). The size of the concave features is diminished in the Δ*psmα* Δ*hld* mutant compared to WT, though they are not entirely abolished (**Figure 7A**). Together, these data suggest that PSM amyloid fibrils deflect the *P. aeruginosa* surfactant layer, resulting in the repulsion of *P. aeruginosa* tendrils. Because surfactant deflection is not completely abolished by the Δ*psmα* Δ*hld* mutant, this suggests that other molecules produced by *S. aureus*, including PSMβ could contribute to surfactant deflection. The deflection of surfactant by *S. aureus* is consistent with the mechanism of tendril repulsion observed by PDMS, TOB-treated *P. aeruginosa*, and PQS (**Figures 5B**, **6A**).

### Repulsion by surfactant produced by other organisms

**Figure 8:**
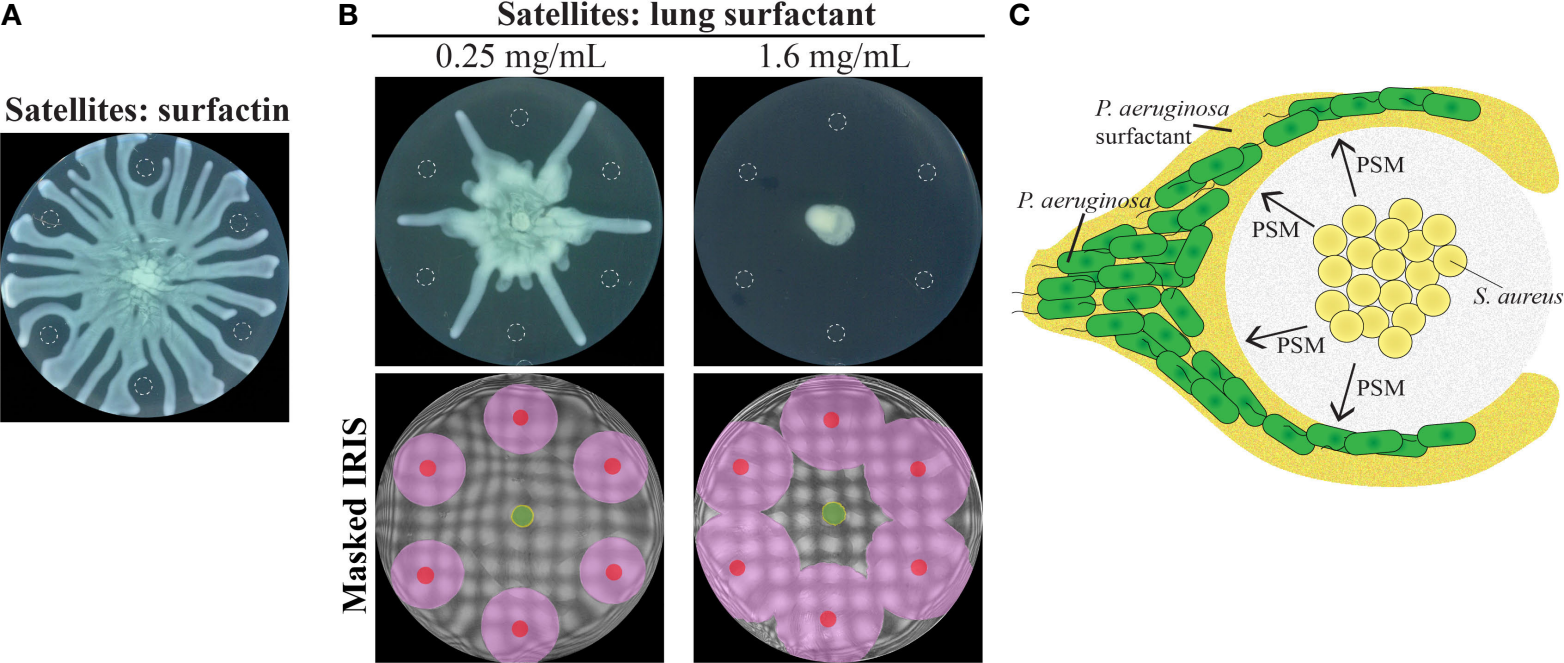
*P. aeruginosa* repulsion mediated by the surfactant layer. **(A)** Swarm interaction assays in which WT *P. aeruginosa* was spotted at the center and 10 µM surfactin from *B. subtilis* was spotted at satellite positions. Images were acquired 16.5 hours following inoculation. **(B)** Swarm interaction assays and masked IRIS images in which WT *P. aeruginosa* was spotted at the center and 6 μL of lung surfactant at the indicated concentrations was spotted at satellite positions. The masked IRIS images indicate the initial inoculum of lung surfactant (red), the fluidic boundary that expands from the lung surfactant (pink), *P. aeruginosa* surfactant (yellow), and *P. aeruginosa* (green). Images of the interaction assays and IRIS images were acquired 18 and 3 hours following inoculation, respectively. The raw IRIS images that do not contain masks are in **Figure S7C** in the **Supplementary Materials**. Dashed lines in **(A, B)** indicate the boundaries of the initial inoculum. **(C)** Schematic of the proposed *P. aeruginosa* swarm repulsion model. PSMs surround the *S. aureus* and deflect the *P. aeruginosa* surfactant layer, thereby causing tendril repulsion.

Our data suggests that molecules that impact the surfactant layer could alter tendril movement. We investigated the potential for surfactants produced by other species to alter tendril patterns. We rationalized that such surfactants would alter the flow of the *P. aeruginosa* surfactant layer. We assessed the impact of surfactin, which is a surfactant that is produced by *B. subtilis*, and of porcine lung surfactant. Consistent with our model, surfactin repelled *P. aeruginosa* swarm tendrils (**Figure 8A**). The effect of lung surfactant on tendrils was dependent on concentration. Lung surfactant at 0.25 mg/mL repelled tendrils but at a higher concentration of 1.6 mg/mL, inhibited *P. aeruginosa* swarm expansion altogether (**Figure 8B** and **Figure S7C** in **Supplementary Materials**). Consistent with the surfactant deflection model, lung surfactant produced a fluidic boundary that expanded radially from the initial spot (**Figure 8B** and **Figure S7C** in **Supplementary Materials**), similar to the boundaries produced by oleic acid and linoleic acid (**Figure S6D**). These results suggest that interactions between the surfactant layer and surfactant molecules have a significant impact on the organization of *P. aeruginosa* swarms.

## Discussion

A fundamental aspect of multispecies bacterial communities is how one organism interacts with another. Here, we have investigated the spatial interaction between two competing opportunistic pathogens of major clinical importance, *P. aeruginosa* and *S. aureus*. Swarms of *P. aeruginosa* were repelled by *S. aureus*, which created a cell-free physical barrier and facilitated coexistence between the two species. We found that physicochemical interactions between the surfactant produced by *P. aeruginosa* and *S. aureus* PSMs create the separation. Our results provide insight into *P. aeruginosa* – *S. aureus* interactions and into *P. aeruginosa* swarming. The findings have important implications on the understanding of how bacteria interact with other species and host environments.

### Differential responses to antibiotic treatment

Our survey of clinical isolates revealed that under TOB-induced stress, *P. aeruginosa* and *S. aureus* have different effects on approaching *P. aeruginosa* swarms. Almost all of the TOB-treated *P. aeruginosa* isolates repelled *P. aeruginosa* swarms (**Figures 1E, F**). The converse was true in *S. aureus* strains. While almost all untreated clinical *S. aureus* strains repelled *P. aeruginosa* swarms, TOB treatment of these strains abolished the repulsive effect (**Figures 2D, E**). This suggests that the antibiotic stress response of *P. aeruginosa* induces production of the signaling molecule PQS. However, an analogous stress response in which *S. aureus* produces additional signaling molecules that repel *P. aeruginosa* was not observed.

### Repulsion of *P. aeruginosa* by *S. aureus*

The combination of IRIS and TEM imaging revealed a novel *P. aeruginosa - S. aureus* interaction. IRIS images showed that *S. aureus* colonies produce a fluidic boundary that expands radially from the colony, forming a ring up to 3 mm beyond the colony’s edge after 10 hours of growth. The fluidic boundary is not visible under ambient lighting or with conventional imaging methods that use non-reflective illumination (**Figure 5A**). TEM showed that PSM-dependent fibrils lie within the fluidic boundary region, up to 3 mm away from the colony’s edge (**Figure 3B**). The combination of IRIS and TEM data raises a number of questions regarding how fibrils are formed and transported. It is possible that PSMs could be carried by a fluid and subsequently nucleate into fibrils during transport away from the colony, or that already-formed fibrils could be carried away by a fluid. We point out that the fluidic boundary produced by *S. aureus* is not limited to the growth conditions on swarm media plates here. In particular, a fluidic boundary is also observed around *S. aureus* colonies that are cultured on LB plates (**Figure 7C**), which are used widely to culture this organism.

We consider how PSMs deflect the *P. aeruginosa* surfactant layer. The surfactant consists of RLs and HAAs, which contain long chain hydrophobic domains. It would be expected that the surfactant layer of *P. aeruginosa* would merge with molecules of similar properties, such as the amphiphilic molecule PQS. In fact, RLs solubilize PQS (Calfee et al., 2005). Surprisingly, the surfactant layer is repelled by PQS (**Figure 6A**). One possibility of how an amphiphilic molecule could repel the surfactant layer is that an intermediate layer of water provides a physical barrier between the surfactant layer and amphiphilic molecule. In the case of PSMs, which are amphipathic, a water layer could form between the surfactant and PSM fibrils, which would deflect the surfactant and produce a cell-free zone of repulsion between *S. aureus* and *P. aeruginosa* (**Figure 8C**). An additional factor that could affect surfactant deflection is viscosity. We found that PDMS of lower viscosity (200 cSt) repelled tendrils to a greater extent than higher viscosity (1000 cSt) PDMS (p=0.02) (**Figure S5B** in **Supplementary Materials**). Thus, a change in the viscosity of the area outside of the *S. aureus* colony produced by PSMs could contribute to surfactant layer deflection. In addition, small differentials in the amphipathicity between the surfactant layer and PSMs could also contribute to deflection. Finally, *S. aureus* may produce additional molecules that disrupt the surfactant layer that have not been identified in this study.

Previous investigations of *P. aeruginosa* - *S. aureus* interactions suggest that *P. aeruginosa* is predisposed to interact with *S. aureus* if the two species are within close proximity (microns) of one another (Limoli et al., 2019). During chronic infections, *P. aeruginosa* and *S. aureus* can evolve a mutualistic relationship in which *P. aeruginosa* does not inhibit *S. aureus* growth, but rather the interaction enhances *P. aeruginosa* growth (Michelsen et al., 2014; Frydenlund Michelsen et al., 2016). In contrast, our study shows that at the longer (millimeter) scale, *S. aureus* repels *P. aeruginosa* and the two species remain isolated, promoting species heterogeneity. In particular, RLs interact with the *S. aureus* membrane, making it more permeable to TOB, and thus sensitize *S. aureus* to TOB-mediated killing (Yarlagadda and Wright, 2019). The separation mechanism described here creates a physical barrier that reduces the likelihood that RLs reach *S. aureus* at the millimeter scale.

### Role of surfactant flow in swarm organization

Our analysis of *P. aeruginosa* swarming suggests that fluid mechanics has a significant role in tendril formation. In particular, the surfactant and tendrils form two separate layers that are coupled in motion in which the former is required for the movement of the latter. The surfactant layer constrains the movement of the swarm tendrils such that *P. aeruginosa* cannot move beyond the surfactant layer boundary. The movement of the surfactant layer around repulsive molecules such as PQS and PSMs thus directs the motion of the tendrils to move around these molecules. Recent work using shadowgraphy shows that RLs penetrate and flow through the agar layer (Deforet, 2023). Our observation of the surfactant flow using IRIS is consistent with movement of RLs through the agar layer. The details of how repulsive molecules affect RL flow within the agar layer are unclear. It is possible that such molecules repel the movement of RLs within the agar or that alternatively, only RLs near the agar surface are affected. In particular, the fluidic boundaries observed moving away from *S. aureus*, oleic acid, linoleic acid, and lung surfactants are consistent with the penetration and movement of these molecules through the agar layer as well. The details of surfactant flow in three dimensions will need to be considered in future studies to address this issue. The mechanism of surfactant disruption has important implications for understanding how tendrils move on surfaces and are repelled or merge with other tendrils, such as is observed with the Δ*sadB* and Δ*rhlC* mutants (Caiazza et al., 2005). The flow of surfactant may need to be considered in such cases and in other aspects of swarm development.

While our results suggest an important role of the surfactant layer in tendril development, they do not preclude a role for sensing. Multiple *P. aeruginosa* sensors can explain the detection of molecules such as RLs and PQS by swarm tendrils. For example, it is possible that sensing of PSMs and PQS could alter the motion of tendrils around *S. aureus* and TOB-treated *P. aeruginosa*, respectively. A mechanism that explains how the tendril motion is altered in response to sensing is lacking, however. Metabolic sensing of hydrocarbon molecules provides an additional possible mechanism that could alter tendril development (Kang et al., 2010). In addition, we note that coupling between the surfactant and tendril layers is likely not the only mechanism that directs tendril movement. For example, after the surfactant layer reaches the boundary of the Petri dish, tendrils continue to move, although at a slower rate. Continued tendril growth in which the surfactant and tendril layers are decoupled may be possible. Biophysical models of pressure-driven flow and Marangoni flow (Fauvart et al., 2012; Yang et al., 2017) may explain the continued expansion of swarm tendrils in the absence of surfactant flow.

### Potential role of surfactant interactions in coexistence

In infection settings, *P. aeruginosa* and *S. aureus* frequently colonize the same environment. Here, it may be mutually beneficial for both species to remain spatially separated. The interactions between *P. aeruginosa* and other organisms may be affected by the presence of surfactant. Similarly, interactions between *P. aeruginosa* and *S. aureus* may be affected by PSM production. Consistent with surfactant and PSMs having important roles in infection, significant levels of RLs have been measured in the lungs of cystic fibrosis patients (Kownatzki et al., 1987). Likewise, PSMs have a major role in lung infection models of *S. aureus* (Bloes et al., 2017). The repulsive interactions observed in this study could facilitate the coexistence of the two species in different micro-niches during coinfection. In particular, PSM fibrils could reduce RL-induced aminoglycoside sensitivity. As the role of swarming in infection is unclear, future experiments will need to address the role of surfactant interactions with PSMs *in vivo*.

The finding that the *P. aeruginosa* surfactant layer has a major role in swarm organization may have implications on *P. aeruginosa* colonization in natural and host environments. The findings suggest that molecules that interact with *P. aeruginosa* surfactants can impact the spatial organization of the population. Moreover, the physicochemical nature of this interaction suggests that chemically-diverse hydrophobic molecules could alter this spatial organization. In support of this claim, surfactin from *B. subtilis* repelled *P. aeruginosa* swarms. This observation is remarkable because *B. subtilis* is evolutionarily distant from *P. aeruginosa* yet causes repulsion of *P. aeruginosa* tendrils. In host environments such as the human lung, cells produce a number of surfactants (Han and Mallampalli, 2015) that could impact colonization or swarm-dependent spreading of *P. aeruginosa* during infection. Our results show that lung surfactant repelled *P. aeruginosa* tendrils and at a sufficiently high concentration, inhibited swarming and the proliferation of *P. aeruginosa*. In contrast, the lack of surfactant facilitated the expansion of *P. aeruginosa*. These findings are consistent with the finding that deficiencies in surfactant production promote susceptibility to *P. aeruginosa* colonization. Indeed, cystic fibrosis patients, who are deficient in lung surfactant production, and mice that are deficient in surfactant production have increased susceptibility to *P. aeruginosa* colonization (Griese et al., 1997; Glasser et al., 2008). Future studies should address the extent that surfactant interactions can alter the expansion of *P. aeruginosa in vivo.* Given that numerous bacterial species found in natural and host environments produce unique surfactants, our findings raise the possibility that surfactants produced among different species of bacteria in polymicrobial environments could have a significant impact on bacterial spatial organization and dissemination, and on disease development.

## Materials and methods

### Growth conditions and materials

Strains were streaked from frozen stocks onto LB Broth-Miller (BD, Franklin Lakes, NJ) plates containing 2% Bacto agar (BD) and incubated overnight at 37°C. Single colonies were inoculated into the same broth and incubated 16 to 18 hours in a roller drum at 20 rpm and 37°C. All strains used in this study are described in **Table S1** in the **Supplementary Materials**. Clinical isolates were obtained from the Whiteson Lab and UCI Health Medical Microbiology Laboratory; no identifying information was collected and therefore IRB approval was not required. The clinical strains used in the study were all of airway and skin wound isolates that were available to us.

Compounds in swarm interaction assays including 200 cSt PDMS, 1000 cSt PDMS, oleic acid, linoleic acid, glyceryl trioleate, glyceryl trilinoleate, Triton X-100, Tween-20, 2-heptyl-3-hydroxy-4-quinolone (PQS), and surfactin isolated from *Bacillus subtilis* (Cat. #S3523), were obtained from Sigma Aldrich (St. Louis, MO) and were used in their pure concentrated forms unless otherwise specified. PQS was dissolved in dimethylsulfoxide (DMSO). Strains at satellite positions were treated with antibiotics using tobramycin (Thermo Fisher Scientific Hampton, NH) at a concentration of 0.5 mg/mL. The porcine pulmonary surfactant extract Curosurf (poractant alfa) (Chiesi USA, Inc., Cary, NC) was obtained at a concentration of 80 mg/mL, diluted in water, and used at the indicated concentrations.

### Swarm interaction assays

Assays were performed as described previously (Bru et al., 2019; Bru et al., 2020). Briefly, 100 x 15mm Petri dishes contained 20 mL of M8 minimum medium (Bru et al., 2020) supplemented with 1 mM MgSO_4_, 0.2% glucose, 0.5% Casamino Acids (BD), and 0.5% Bacto agar (BD). Following sterilization, media in Petri dishes solidified for 1 hour with lids on at room temperature, and then dried for 30 minutes without lids in a laminar flow hood at 300 cubic ft./min. with approximately 40 to 50% ambient humidity. Strains were cultured 16 to 18 hours in LB broth. WT *P. aeruginosa* was spotted at the center of the swarming plate in a 5 µL droplet. Strains or compounds were spotted at satellite positions using 6 µL droplets at 6 satellite positions that were equidistant from each other along a 5.8 cm-diameter circle centered at the swarming plate (**Figure S7D** in the **Supplementary Materials**). Plates were incubated overnight at 37°C with a darkened Petri dish lid. Images were acquired every 30 minutes for 18 to 20 hours using an Epson scanner (Epson, Los Alamitos, CA) that was controlled using RoboTask (robotask.com) and then processed using ImageJ 1.54d (NIH, Bethesda, MD). For the antibiotic treatment of strains at satellite positions, tobramycin (Thermo Fisher Scientific) was mixed with the bacterial inoculum to a final concentration of 0.5 mg/mL and the mixture was spotted immediately onto swarming plates in 6 µL droplets. The repulsion radius at each satellite colony was measured as the distance from the center of the satellite to the nearest tendril along a line that is tangent to the 5.8 cm-diameter circle that is concentric with the swarming plate (**Figure S7D** in the **Supplementary Materials**). If a tendril contacted the boundary of the initial satellite spot, the repulsion radius was marked as zero, except for the measurements to determine the correlation between repulsion radius and surfactant deflection area by PQS (**Figure 6B**).

### Transmission electron microscopy

Mesh copper grids that were 3 mm in diameter (Electron Microscopy Sciences, Hatfield, PA) were placed with the carbon layer facing up at or adjacent to inoculation positions on swarming plates. Six microliters of *S. aureus* that had been cultured in LB broth for 16 to 18 hours was spotted onto the inoculum position. Plates containing the inoculums and copper grids were incubated at 37°C and 50% humidity for 16 to 18 hours. The grids were stained using 1% uranyl acetate (Electron Microscopy Sciences), dried overnight at room temperature, and imaged using a JEM-2800 High Throughput Electron Microscope (JEOL, Akishima, Japan) using an acceleration voltage of 200 kV.

### Contact angle measurements

Test compounds were pipetted vertically in one microliter droplets at room temperature onto a Notak oleophobic-coated (SilcoTek, Bellefonte, PA) polished stainless steel sheet surface and imaged immediately for 10 seconds using a Model 90 goniometer (ramé-hart, Succasunna, NJ) with a Summit SK2-3.1X 3.1MP digital camera (OptixCam, Roanoke, VA). Contact angles were measured from timelapses at the 7 second timepoint after the initial droplet placement onto the surface using ImageJ and the sessile drop method. Angles were measured for the left and right sides of the droplet in the images and averaged.

### Surface growth quantification

The growth of *S. aureus* on swarm plates (**Figure S3A** in **Supplementary Materials**) was determined by acquiring images as described in the swarm interaction assays. Surface growth intensities were computed using ImageJ by measuring the pixel intensities of *S. aureus* colonies along a ring that corresponded to *S. aureus* growth along the outer radius of the colony. Pixel intensities of *S. aureus* were subtracted by those outside of the *S. aureus* colony, arriving at a surface growth intensity. Intensities were normalized by dividing by the value measured within 1 hour of inoculation for each strain.

### IRIS imaging

Imaging was performed as described previously (Kasallis et al., 2023). Briefly, swarming plate surfaces were illuminated by an ASUS LCD VE278Q monitor (ASUS, Fremont, CA) that displayed a structured image that consisted of evenly-spaced white squares in a 17 x 31 grid pattern on a black background (**Figure S6A** in **Supplementary Materials**). The reflection of the structured image on the swarming plate surface was acquired using a Canon EOS Rebel T7 DSLR (Canon, Melville, NY) with a Canon EF-S 18-55 mm lens. The inner surfaces of Petri dishes were scratched with a metal wire brush to reduce reflections. Swarming plates were incubated at 37˚C and 50% humidity. For timelapses, the lid was removed and replaced at regular intervals using a robotic arm to allow for image acquisition. Masks of the IRIS images were constructed by passing the magnetic lasso tool in Adobe Photoshop (Adobe, San Jose, California) over edge features in IRIS images to identify boundaries. Zones that were enclosed within the boundaries were designated as *P. aeruginosa* or surfactant. Differential images were constructed using the imabsdiff function in Matlab version R2017a (Mathworks, Natick, MA) using images that were acquired at 5 minute (**Figure 5A**) or 30 minute intervals (all others). The surfactant deflection area was defined as the total area in the vicinity of the satellite spot that did not contain *P. aeruginosa* surfactant (**Figure S7B** in the **Supplementary Materials**). Our identification of the *P. aeruginosa* surfactant layer boundary was aided by following surfactant flow in timelapse IRIS movies and using differential IRIS images. An additional boundary of the deflection area was defined as an arc that connected two of the nearest surfactant layer boundaries that were not deflected.

## Data availability statement

The raw data supporting the conclusions of this article will be made available by the authors, without undue reservation.

## Author contributions

AS, J-LB, and QZ developed the IRIS device. J-LB, SJK, KW, NMH-K, EW, DHL, and AS designed experiments and determined project direction. J-LB, SJK, RC, QZ, PP, and JN performed experiments and analyzed data. J-LB, SJK, and AS drafted and edited the manuscript. All authors contributed to the article and approved the submitted version.
