# Supplementary material for: The great divide: rhamnolipids mediate separation between P. aeruginosa and S. aureus
Source: Front Cell Infect Microbiol. 2023 Sep 15;13:1245874. doi: 10.3389/fcimb.2023.1245874 (PMC10540625; doi:10.3389/fcimb.2023.1245874)
Supplement: Supplementary file 1 [file DataSheet_1.pdf]

Supplementary Materials

The great divide: rhamnolipids mediate separation between *P. aeruginosa* and *S. aureus*

Jean-Louis Bru†, Summer J. Kasallis†, Rendell Chang, Quantum Zhuo, Jacqueline Nguyen, Phillip Pham, Elizabeth Warren, Katrine Whiteson, Nina Molin Høyland-Kroghsbo, Dominique H. Limoli, Albert Siryaporn*

†These authors contributed equally to this work and share first authorship

*To whom correspondence should be addressed: asirya@uci.edu

Supplementary Movie Legends
Supplementary Figures S1-S7
Supplementary Table S1
Supplementary References

Supplementary Movie Legends

Movie S1. Impact of tobramycin on *S. aureus* growth. Swarm interaction assays in which wild-type *P. aeruginosa* was spotted at the center and *S. aureus* clinical isolate 5 (CISa-5) was spotted at satellite positions with tobramycin (TOB) (right) or without TOB (left). Tobramycin treatment was performed by mixing TOB with bacteria to a final concentration of 0.5 mg/mL and spotting 6 μL of the mixture onto the swarm plate. Images were captured every 30 minutes following inoculation over the course of 18 hours.

Movie S2. *P. aeruginosa* swarm interaction assays with 1000 cSt PDMS at satellite positions. IRIS (left) and differential IRIS (right) timelapses of swarm interaction assays in which *P. aeruginosa* was spotted at the center and 1000 cSt PDMS was spotted at satellite positions. Images were captured every 30 minutes following inoculation over the course of 18 hours.

Movie S3. *P. aeruginosa* swarm interaction assays with tobramycin-treated *P. aeruginosa* at satellite positions. IRIS (left) and differential IRIS (right) timelapses of swarm interaction assays in which *P. aeruginosa* was spotted at the center and tobramycin (TOB)-treated *P. aeruginosa* was spotted at satellite positions. Treatment was performed by mixing TOB with bacteria to a final concentration of 0.5 mg/mL and spotting 6 μL of the mixture onto the swarm plate. Images were captured every 30 minutes following inoculation over the course of 18 hours.

Movie S4. *P. aeruginosa* swarm interaction assays with *P. aeruginosa* Δ*rhlAB* at satellite positions. IRIS (left) and differential IRIS (right) timelapses of swarm interaction assays in which wild-type *P. aeruginosa* and the *P. aeruginosa* Δ*rhlAB* mutant were spotted at the center and satellite positions, respectively. Images were captured every 30 minutes following inoculation over the course of 18 hours.

Movie S5. *P. aeruginosa* swarm interaction assays with PQS at satellite positions. IRIS (left) and differential IRIS (right) timelapses of swarm interaction assays in which wild-type *P. aeruginosa* was spotted at the center and PQS at the concentrations indicated in mM was spotted at the satellite positions. Images were captured every 30 min following inoculation over the course of 18 hours.

Movie S6. *P. aeruginosa* swarm interaction assays with *S. aureus* at satellite positions. IRIS (left) and differential IRIS (right) timelapses of swarm interaction assays in which wild-type *P. aeruginosa* was spotted at the center and wild-type *S. aureus* strain USA300 s1 was spotted at satellite positions. Images were captured every 30 minutes following inoculation over the course of 18 hours.

Supplementary Figures S1-S7

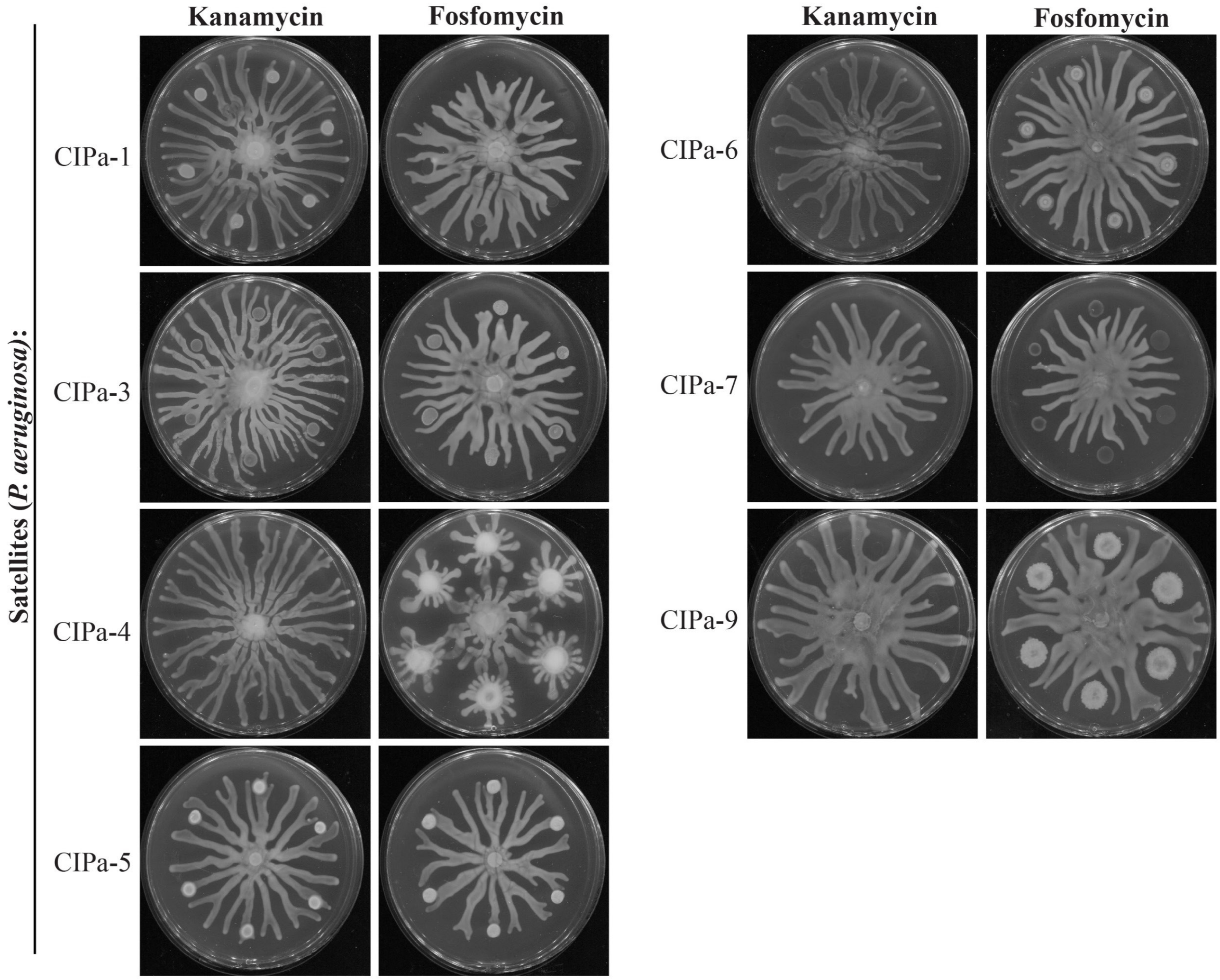

Figure S1. Antibiotic resistance among *P. aeruginosa* clinical isolates. Swarm interaction assays in which wild-type *P. aeruginosa* was spotted at the center and clinical isolates of *P. aeruginosa* (CIPa) were treated with either kanamycin or fosfomycin, and spotted at satellite positions. Antibiotic treatment was performed by mixing bacteria with antibiotics to a final concentration of 25 mg/mL for kanamycin or 40 mg/mL for fosfomycin, and spotting 6 μL of the mixture onto the swarm plate. Images were acquired 16 to 18 hours following inoculation.

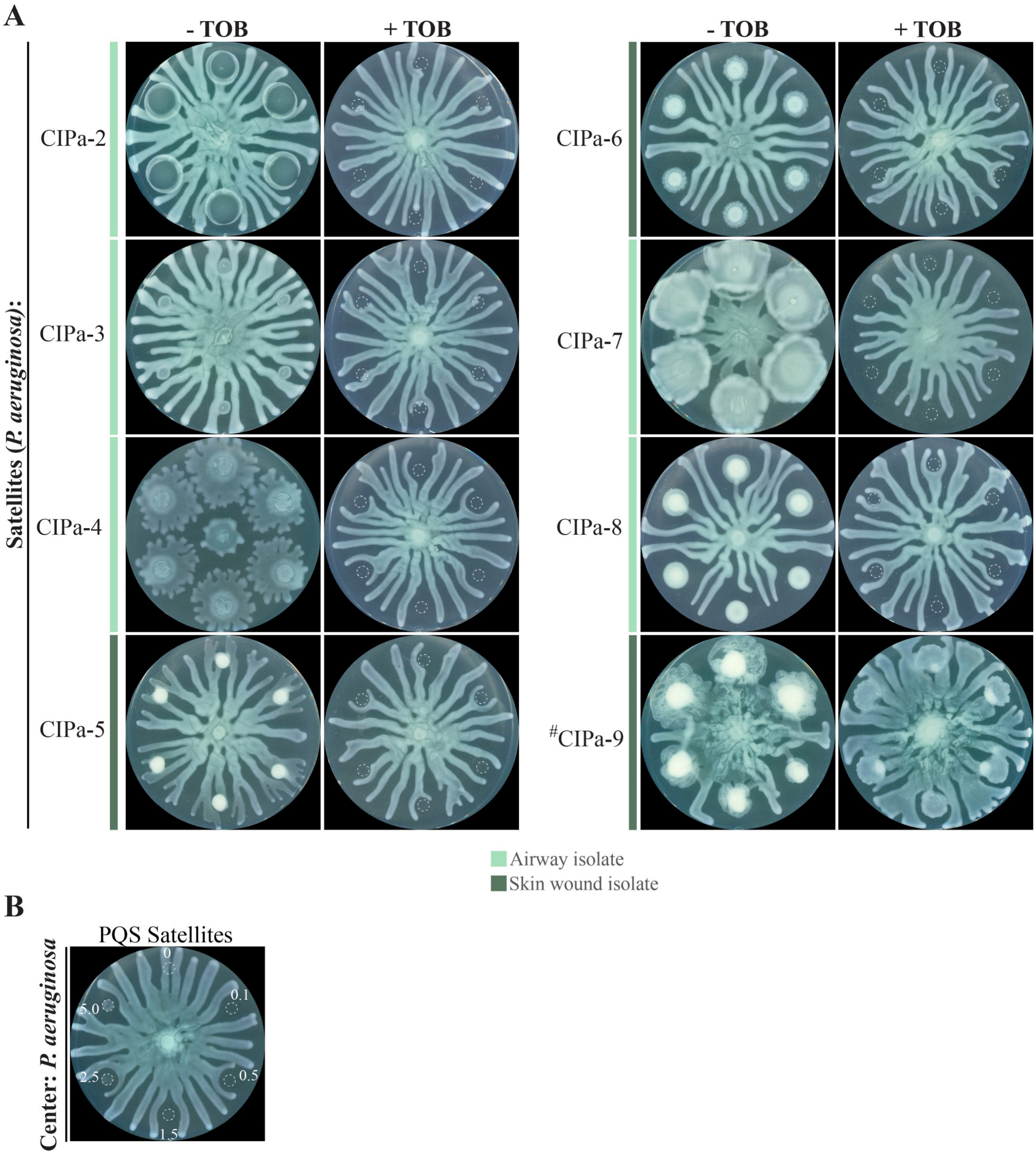

Figure S2. Swarm repulsion by clinical isolates of *P. aeruginosa*. (A) Swarm interaction assays in which wild-type *P. aeruginosa* was spotted at the center and CIPa strains were spotted at satellite positions with or without tobramycin (TOB). Tobramycin treatment was performed by mixing TOB with bacteria to a final concentration of 0.5 mg/mL and spotting 6 μL of the mixture onto the swarm plate. Color bars indicate if the strains were isolated from the airway or skin wound. #CIPa-9 was resistant to TOB. (B) Swarm interaction assay in which wild-type *P. aeruginosa* was spotted at the center and PQS at the indicated concentrations in mM was spotted at satellite positions. Dashed lines indicate the boundaries of initial inoculum spots. Images were acquired 18 to 20 hours following inoculation.

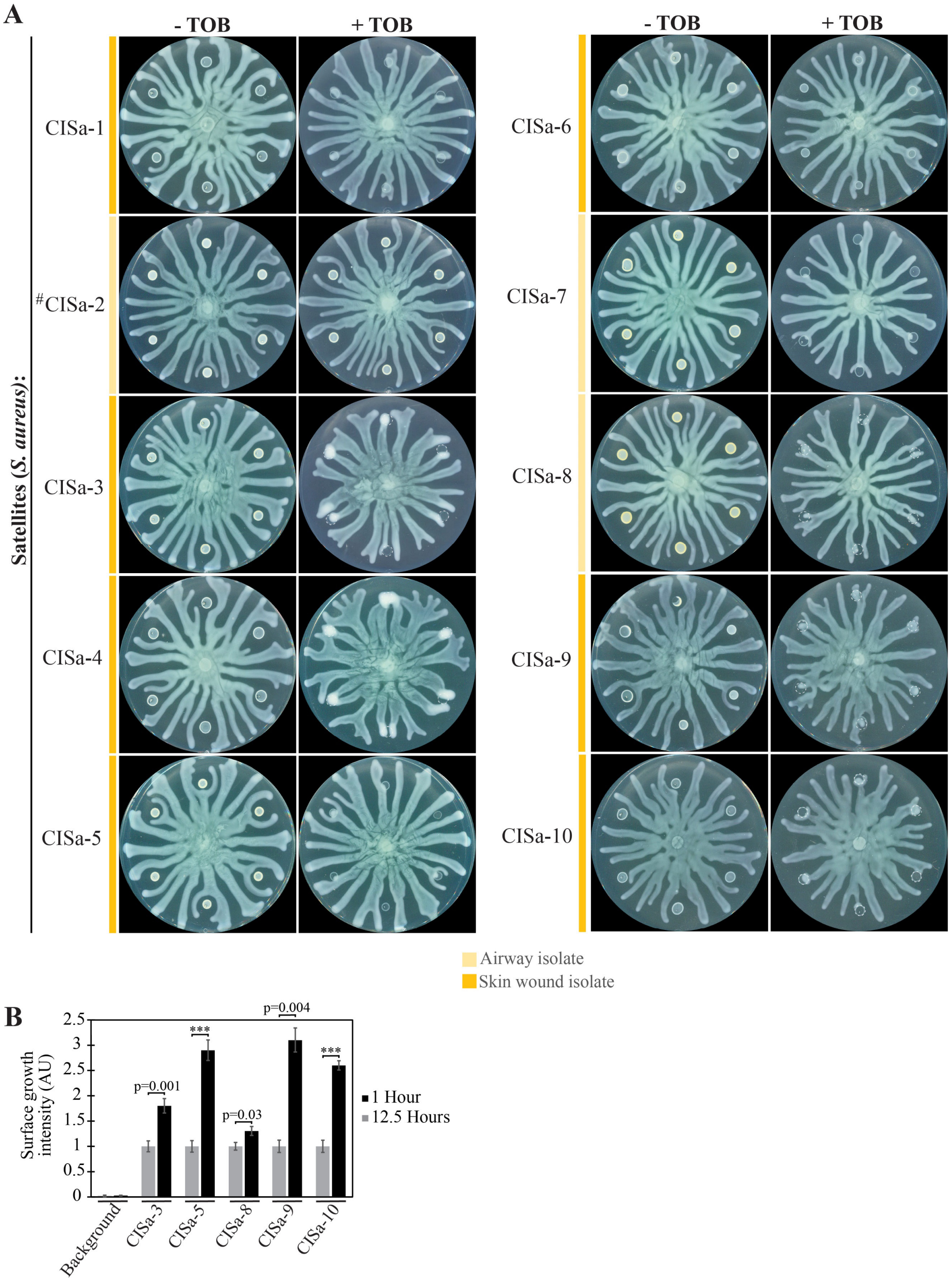

Figure S3. Swarm repulsion by clinical isolates of *S. aureus*. (A) Swarm interaction assays in which wild-type *P. aeruginosa* was spotted at the center and clinical isolates of *S. aureus* (CISa) were spotted at satellite positions with or without tobramycin (TOB). Tobramycin treatment was performed by mixing TOB with bacteria to a final concentration of 0.5 mg/mL and spotting 6 µL of the mixture onto the swarm plate. Color bars indicate if the strains were isolated from the airway or skin wound. #CISa-2 was resistant to TOB. Images were acquired 18 to 20 hours following inoculation. (B) Quantification of surface growth for CISa strains for which the surface growth is not apparent in the TOB-treated colonies in (A). Surface growth intensities were measured within 1 hour and at 12.5 hours following inoculation. Bars represent the surface growth intensities from at least three satellite positions and error bars indicate standard error. T-tests were performed using two-tailed distributions with unequal variance. *** denotes $p < 0.001$.

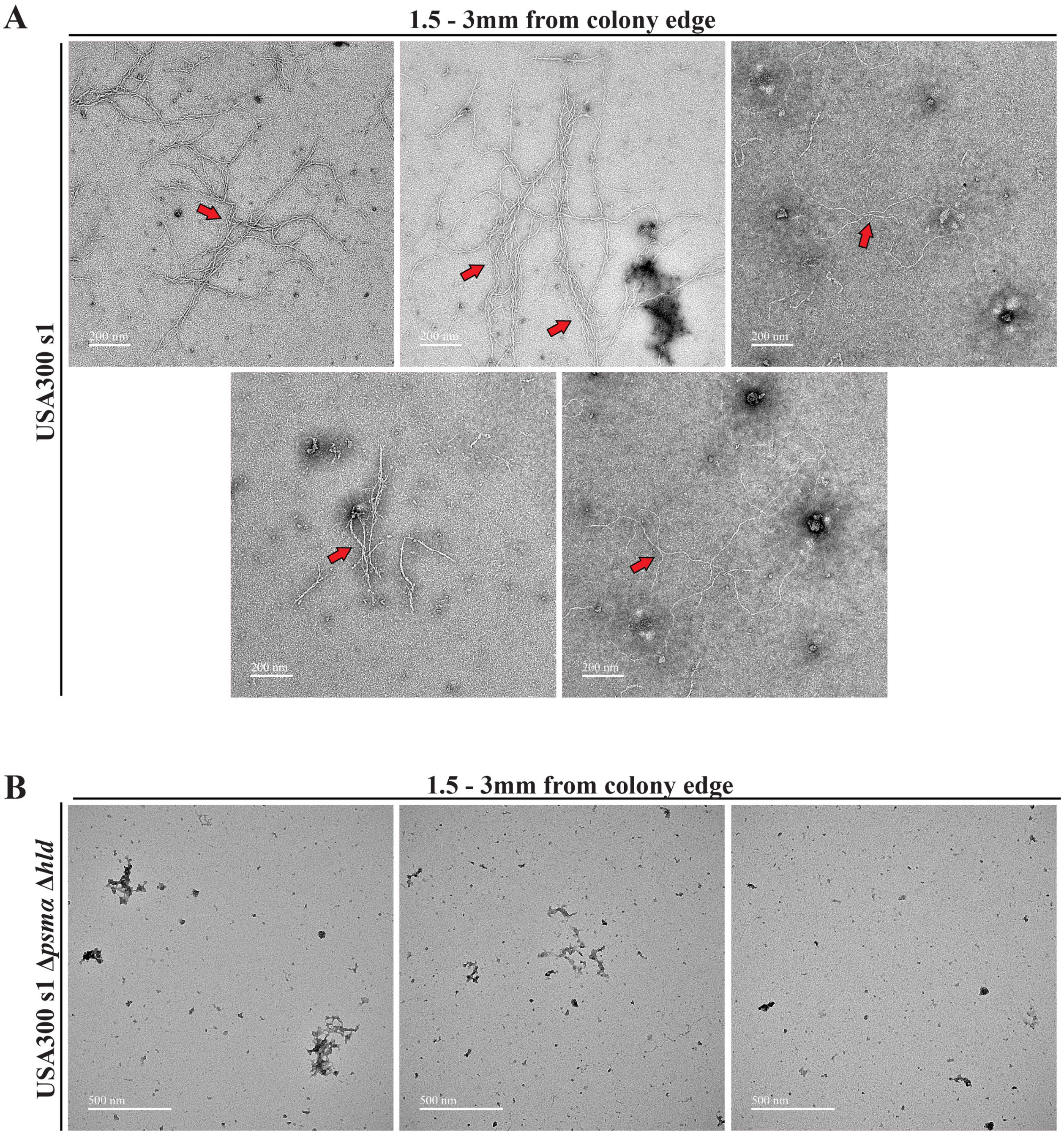

Figure S4. Transmission electron microscopy (TEM) images in the vicinity of *S. aureus* colonies. TEM images of areas that were between 1.5 and 3mm from the edge of colonies of (A) wild-type *S. aureus* strain USA300 s1 and (B) the *S. aureus* USA300 s1 Δ*psmα* Δ*hld* mutant. Scale bars indicate 200 nm in (A) and 500 nm in (B).

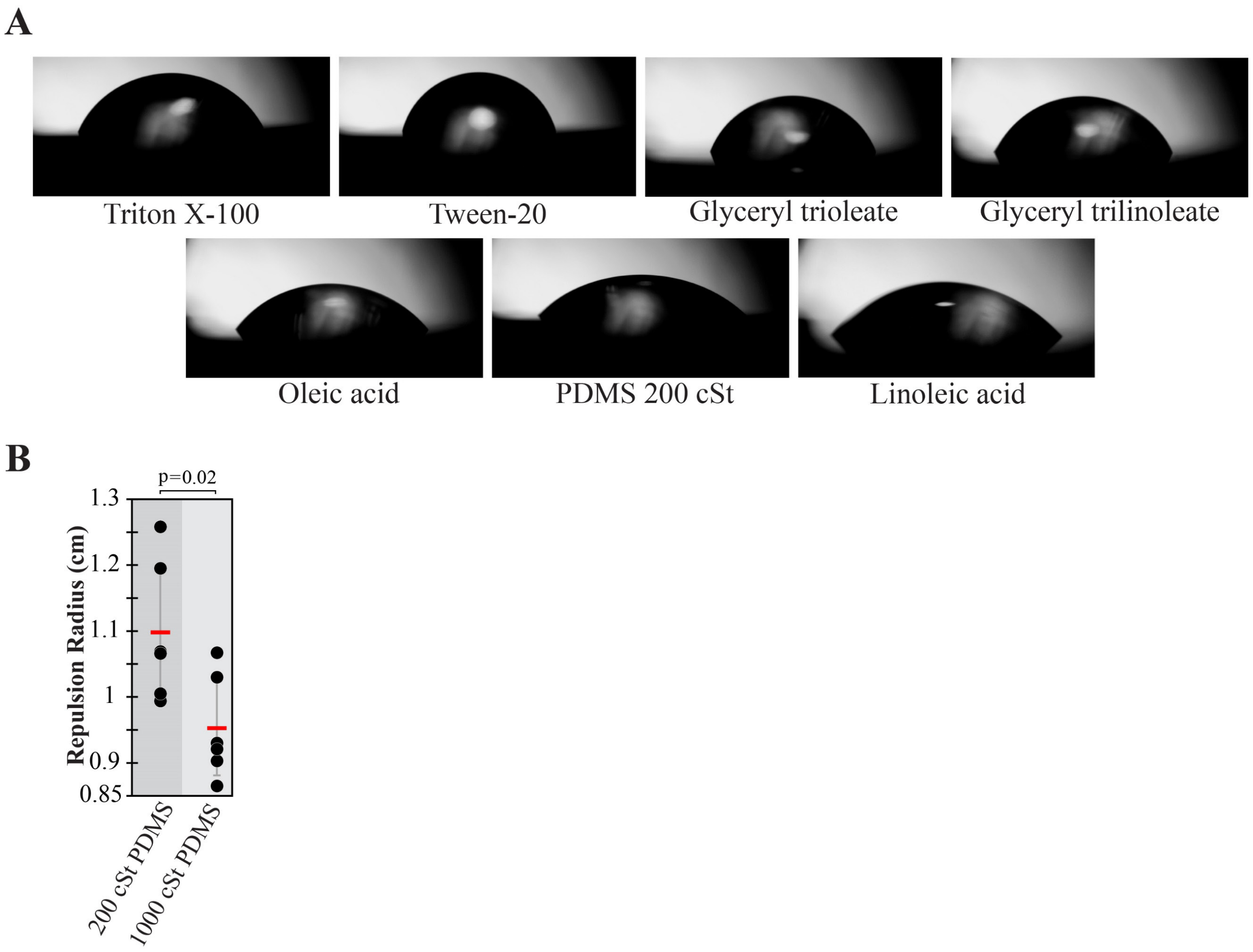

Figure S5. Contact angles and repulsion radii of hydrophobic molecules. (A) Images of hydrophobic molecules on an oleophobic surface acquired by a contact angle goniometer. Triton X-100 and Tween-20 were used at concentrations of 0.2% and 2%, respectively. (B) Tendril repulsion radii by 200 cSt and 1000 cSt viscosities of PDMS. Measurements were performed on images that were acquired 15 hours following inoculation. Red lines indicate average repulsion radius and error bars indicate standard deviation. The t-test was performed as a two-tailed distribution with unequal variance.

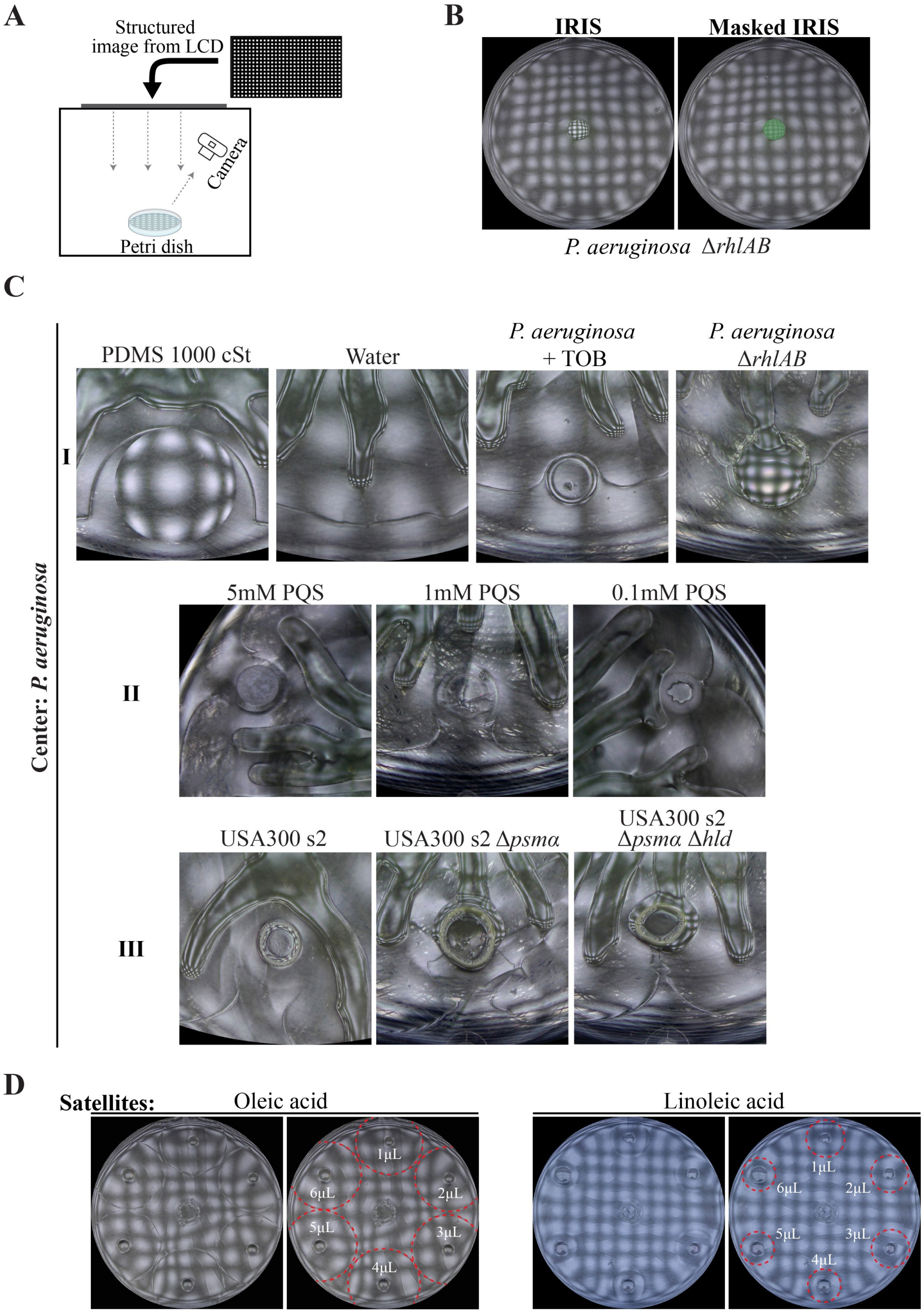

Figure S6. Surfactant production and surfactant interactions. (A) Schematic of the Imaging using Reflected Illuminated Structures (IRIS) setup. A structured image is projected onto a Petri dish and the image reflected from the surface is captured using a camera. (B) IRIS image (left) and masked IRIS image (right) of *P. aeruginosa* Δ*rhlAB* (green) spotted at the center of the plate and imaged after 20 hours of growth. A surfactant boundary was not detected. (C) IRIS images in which wild-type *P. aeruginosa* was spotted at the center and test strains or compounds were spotted at satellite positions. The images in I, II, and III were processed to produce the masked IRIS images in Figs. 5B, 6A, and 7A, respectively. (D) IRIS images in which wild-type *P. aeruginosa* was spotted at the center and oleic acid or linoleic acid were spotted at satellite positions. The volumes that were spotted and fluidic boundaries are indicated (red dashed lines) in the images to the right. Images were acquired 5 hours following inoculation.

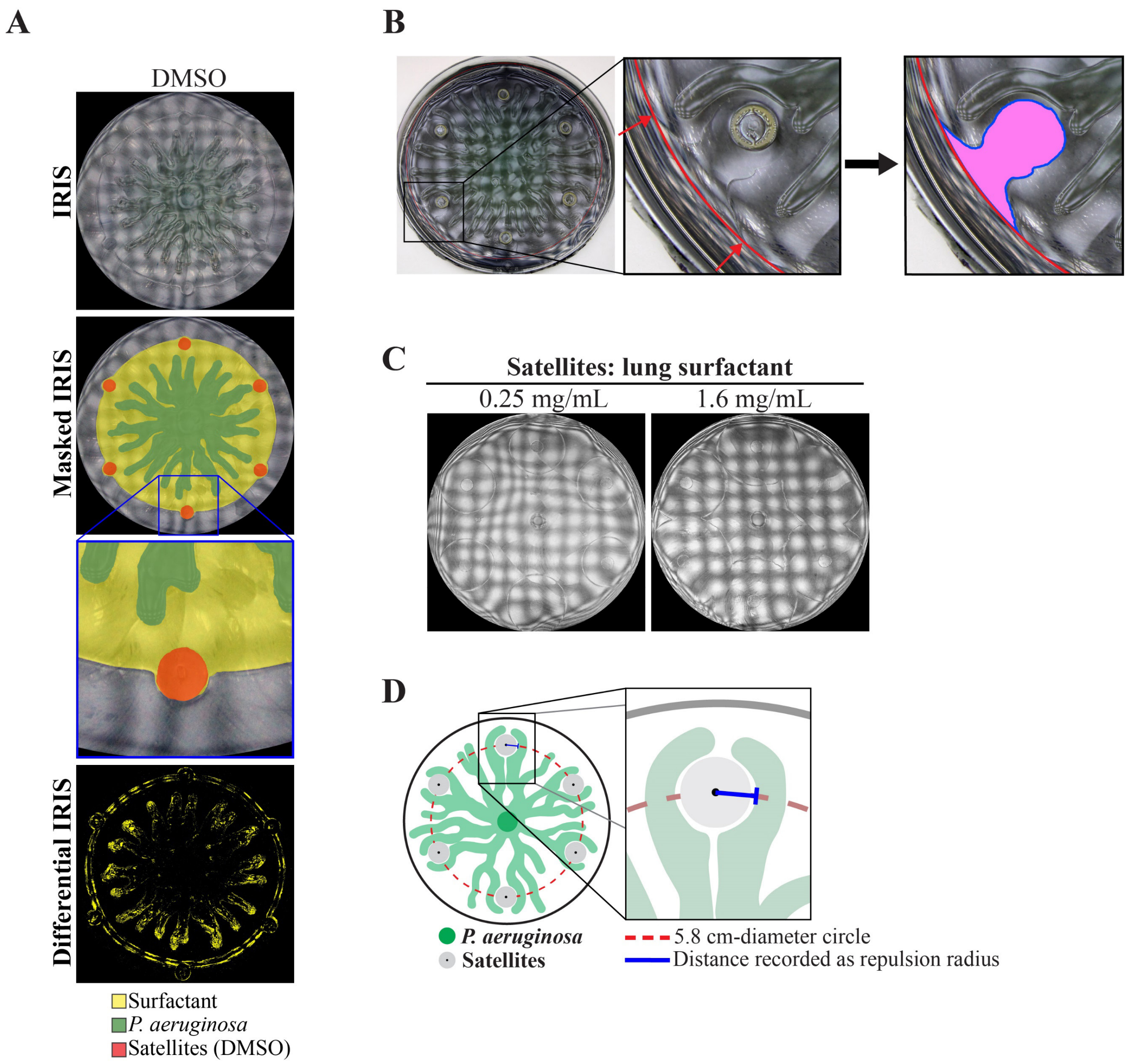

Figure S7. Swarm interactions with DMSO and surfactant, and tendril measurement methods. (A) IRIS, masked IRIS, and differential IRIS images of a swarm interaction assay in which *P. aeruginosa* was spotted at the center and DMSO was spotted at satellite positions. Images were acquired 14 hours following inoculation. Masked IRIS images indicate the surfactant layer (yellow), *P. aeruginosa* (green), and the initial boundaries of the satellite spots (red). The differential IRIS image indicates components of the surfactant layer and *P. aeruginosa* that are dynamic. (B) Graphic depicting how the surfactant deflection area was determined, using *S. aureus* USA300 s1 at the satellite position as an example. The boundary of the surfactant layer near the satellite position was identified (blue line). An arc (red) that connects two of the nearest surfactant layer boundaries that were not deflected was defined as an additional boundary. The surfactant deflection area (pink) was defined as the area enclosed by these boundaries. (C) IRIS images of a swarm interaction assay in which *P. aeruginosa* was spotted at the center and 6 µL of lung surfactant at the indicated concentrations were spotted at satellite positions. Images were processed to produce the masked IRIS images in Fig. 8B. (D) Schematic depicting how tendril repulsion radius is determined. Test strains or compounds (gray) were spotted along a 5.8 cm-diameter circle (red dashed line) that is concentric with the swarming plate. The repulsion radius (blue line) at each satellite colony was measured as the distance from the center of the satellite position (black dot) to the nearest tendril along a line that is tangent to the circle. If a tendril contacted the boundary of the initial satellite spot, the repulsion radius was recorded as zero.

Supplementary Table S1 – Strains used in this study

Name	Lab Strain Name	Strain	Description	References	Source
CIPa-1	PAnmFLR01 (P1)	*P. aeruginosa*	Airway isolate	(Quinn et al., 2016)	Whiteson lab
CIPa-2	PAmFLR02 (P2m)	*P. aeruginosa*	Airway isolate	(Quinn et al., 2016)	Whiteson lab
CIPa-3	WI 1-2	*P. aeruginosa*	Airway isolate	This study	UCI Health
CIPa-4	WI 4-7	*P. aeruginosa*	Airway isolate	This study	UCI Health
CIPa-5	WI 12-22	*P. aeruginosa*	Skin wound isolate	This study	UCI Health
CIPa-6	WI 14-26	*P. aeruginosa*	Skin wound isolate	This study	UCI Health
CIPa-7	WI 16-30	*P. aeruginosa*	Airway isolate	This study	UCI Health
CIPa-8	WI 17-32	*P. aeruginosa*	Airway isolate	This study	UCI Health
CIPa-9	WI 20-38	*P. aeruginosa*	Skin wound isolate	This study	UCI Health
CISa-1	WI 2-4	*S. aureus*	Skin wound isolate	This study	UCI Health
CISa-2	WI 6-10	*S. aureus*	Airway isolate	This study	UCI Health
CISa-3	WI 7-12	*S. aureus*	Skin wound isolate	This study	UCI Health
CISa-4	WI 9-15	*S. aureus*	Skin wound isolate	This study	UCI Health
CISa-5	WI 10-18	*S. aureus*	Skin wound isolate	This study	UCI Health
CISa-6	WI 12-21	*S. aureus*	Skin wound isolate	This study	UCI Health
CISa-7	WI 13-24	*S. aureus*	Airway isolate	This study	UCI Health
CISa-8	WI 15-28	*S. aureus*	Airway isolate	This study	UCI Health
CISa-9	WI 19-36	*S. aureus*	Skin wound isolate	This study	UCI Health
CISa-10	WI 21-40	*S. aureus*	Skin wound isolate	This study	UCI Health
USA300 s1	SADHL129	*S. aureus*	USA300 (LAC)	(Chaney et al., 2017)	Wozniak lab
USA300 s1 Δ*psmα* Δ*hld*	SADHL130	*S. aureus*	USA300 (LAC) Δ*psmα*1-4 *δ*ATG – ATT Confirmed using colony PCR with the following primers: CTCTATCCAGCTGAGCTACC (forward strand) and CACGATGCCCATCAACTTCC (reverse strand)	(Syed et al., 2015)	Wozniak lab
USA300 s2	SADHL121	*S. aureus*	USA300 (LAC)	(Centers for Disease Control and Prevention (CDC), 2003; McDougal et al., 2003)	Alex Horsewill lab
USA300 s2 Δ*psmα*	SADHL138	*S. aureus*	USA300 (LAC) Δ*psmα*1-4		Alex Horsewill lab
USA300 s2 Δ*psmα* Δ*hld*	SADHL137	*S. aureus*	USA300 (LAC) Δ*psmα*1-4 *δ*ATG – ATT		Alex Horsewill lab
USA300 (JE2)	SADHL43	*S. aureus*	USA300 (LAC) derivative	(Fey et al., 2013)	Cheung lab
Wild-type *P. aeruginosa*	AFS27E.1	*P. aeruginosa*	PA14 *att*Tn7::[$P_{A1/04/03}$-*mCherry*] *aacC1*::FRT	(Bru et al., 2019)	Siryaporn lab
P. aeruginosa Δ(*rhlAB*)	BR04.1	*P. aeruginosa*	PA14 Δ(*rhlAB*)::FRT	(Bru et al., 2019)	Siryaporn lab
P. aeruginosa Δ(*rhlAB*) Δ(*pqsA*)	AFS82.1	*P. aeruginosa*	PA14 Δ(*rhlAB*)::FRT Δ(*pqsA*)::FRT	(Bru et al., 2019)	Siryaporn lab

Supplementary References

Bru, J.-L., Rawson, B., Trinh, C., Whiteson, K., Høyland-Kroghsbo, N. M., and Siryaporn, A. (2019). PQS Produced by the Pseudomonas aeruginosa Stress Response Repels Swarms Away from Bacteriophage and Antibiotics. *J Bacteriol* 201, e00383-19. doi: 10.1128/JB.00383-19.

Centers for Disease Control and Prevention (CDC) (2003). Outbreaks of community-associated methicillin-resistant Staphylococcus aureus skin infections--Los Angeles County, California, 2002-2003. *MMWR Morb Mortal Wkly Rep* 52, 88.

Chaney, S. B., Ganesh, K., Mathew-Steiner, S., Stromberg, P., Roy, S., Sen, C. K., et al. (2017). Histopathological comparisons of Staphylococcus aureus and Pseudomonas aeruginosa experimental infected porcine burn wounds. *Wound Repair Regen* 25, 541–549. doi: 10.1111/wrr.12527.

Fey, P. D., Endres, J. L., Yajjala, V. K., Widhelm, T. J., Boissy, R. J., Bose, J. L., et al. (2013). A Genetic Resource for Rapid and Comprehensive Phenotype Screening of Nonessential Staphylococcus aureus Genes. *mBio* 4, e00537-12. doi: 10.1128/mBio.00537-12.

McDougal, L. K., Steward, C. D., Killgore, G. E., Chaitram, J. M., McAllister, S. K., and Tenover, F. C. (2003). Pulsed-field gel electrophoresis typing of oxacillin-resistant Staphylococcus aureus isolates from the United States: establishing a national database. *J Clin Microbiol* 41, 5113–5120. doi: 10.1128/JCM.41.11.5113-5120.2003.

Quinn, R. A., Phelan, V. V., Whiteson, K. L., Garg, N., Bailey, B. A., Lim, Y. W., et al. (2016). Microbial, host and xenobiotic diversity in the cystic fibrosis sputum metabolome. *ISME J* 10, 1483–1498. doi: 10.1038/ismej.2015.207.

Syed, A. K., Reed, T. J., Clark, K. L., Boles, B. R., and Kahlenberg, J. M. (2015). Staphlyococcus aureus phenol-soluble modulins stimulate the release of proinflammatory cytokines from keratinocytes and are required for induction of skin inflammation. *Infect Immun* 83, 3428–3437. doi: 10.1128/IAI.00401-15.
